# The Active Components of Sunflower (*Helianthus annuus* L.) Calathide and the Effects on Urate Nephropathy Based on COX-2/PGE2 Signaling Pathway and the Urate Transporter URAT1, ABCG2, and GLUT9

**DOI:** 10.3389/fnut.2021.769555

**Published:** 2022-01-10

**Authors:** Huining Dai, Shuai Lv, Zi'an Qiao, Kaiyu Wang, Xipeng Zhou, Chunyang Bao, Shitao Zhang, Xueqi Fu, Wannan Li

**Affiliations:** ^1^Edmond H. Fischer Signal Transduction Laboratory, School of Life Sciences, Jilin University, Changchun, China; ^2^School of Life Sciences, Jilin University, Changchun, China; ^3^Jilin Province Medical Device Inspection Institute, Changchun, China

**Keywords:** sunflower calathide, urate nephropathy, COX-2/PGE2, ABCG2, URAT1, GLUT9

## Abstract

The sunflower (*Helianthus annuus* L.) calathide is gradually used as an alternative treatment for hyperuricemia; nevertheless, evidence regarding its main components and therapeutic capacity for urate nephropathy is lacking. Identification of sunflower calathide aqueous extract (SCE) was rapidly done by UPLC-ESI-Q-Orbitrap, and 32 water-soluble compounds with a comprehensive score >80 were discovered. Besides, yeast extract was administrated to induce high UA levels and hyperuricemic renal injury. We found that SCE treatment not only decreased UA levels to a comparable degree as allopurinol and benzbromarone, but also reduced the BUN levels and participated in kidney injury repair induced by uric acid. Moreover, it regulated the expression of URAT1 and ABCG2, especially inhibiting the GLUT9 in the normal kidney. Results were multifacetedly evaluated with a view to suggesting a possible mechanism of action as compared with those of allopurinol and benzbromarone by western blotting, H&E staining, and immunohistochemistry. However, the H&E staining showed histological changes in model, benzbromarone, and allopurinol groups rather than SCE treatments, and at the same time, the uric acid was identified as a cause of renal damage. The antiinflammatory effects and the regulations of COX-2/PGE2 signaling pathway were revealed on the LPS-induced RAW264.7 cells, indicating that the SCE not only increased cellular proliferation but also downregulated the COX-2, PGE2, NO, and IFN-γ cytokines in the RAW264.7 cells. To conclude, the SCE acts on urate transporters and contributes to prevent urate nephropathy *via* alleviating inflammatory process involving COX-2/PGE2 signaling pathway. It is available to develop SCE as food supplemental applications for hyperuricemia and nephritic inflammation.

## Introduction

Sunflower (*Helianthus annuus* L.) is a member of Asteraceae family, native to North America, and extensively plants in China, Russia, Argentina, and France in recent years (1). It is officially recorded that sunflower calathide (head) has long been used as a folk remedy for hypertension, headache, abdominal pain, and uterine bleeding in *Chinese Pharmacopoeia, Chinese Materia Medica*, and *Great Dictionary of Chinese Medicine* (*The second edition, 2006*). Students from Jilin University have investigated the effects on hyperuricemia and gout; meanwhile, a clinical observation of UA-lowering effect and safety among 70 hyperuricemia patients was obtained (2). Besides, others demonstrated that sunflower head extract was not only able to lower high UA levels and reduce swollen ankles, but also alleviate urate deposition in hyperuricemic rodents (3). The inhibition of xanthine oxidase and adenosine deaminase in hyperuricemia mice were detected as well (4). However, sunflower calathide, which was considered as an agricultural waste, has not been researched as widely as seeds and pectin. Sunflower head pectin has long been used for functional foods (5, 6) and the antioxidant activities of pectin were investigated (7). Natural low-methoxyl pectin extracted from sunflower head could be served as a potential low-calorie or sugar-free food in health care industry (8).

Previous studies have shown that the incidence of urate nephropathy caused by hyperuricemia (HUA) is increasing (9). Approximately 60–70% of UA is eliminated and filtered by glomerular filtration. HUA is a metabolic disease caused by decreased excretion or increased production of UA, which is the end product of purine metabolism in human due to the uricase absence, and is associated with metabolic syndrome, diabetes, hypertension, and kidney disease (10, 11). A previous study indicated that chronic kidney disease (CKD) is accompanied with hyperuricemia, so that it is necessary to lower the high UA levels and prevent CKD deterioration (12); however, the mechanism by which high levels of UA increase the incidence of kidney injury remains unknown.

Benzbromarone, a small molecule used to treat primary hyperuricemia, promotes UA excretion by inhibiting URAT1 transporter in hyperuricemia patients (13). Allopurinol is another hyperuricemia therapy which inhibits xanthine oxidase activity, preventing the conversion of hypoxanthine and xanthine to UA (14). However, these medicines have side effects and cannot be used for a long-term treatment of hyperuricemia. In addition to change lifestyle, treatment is usually required for hyperuricemia, and on account of the medication side effects, medicinal and natural herbals, such as Chinese medicine, would be a better option for the alternative therapies.

Diverse urate transporters in the kidneys are involved in the regulation of serum UA levels. The urate transporter 1 (URAT1), encoded by the SLC22A12 gene, is expressed at the brush border of the renal proximal tubule epithelial cells and is the major transporter for renal urate reabsorption (15–17). This makes it an important target for hyperuricemia research. In addition, the ATP binding cassette transporter member 2 of subfamily G (ABCG2) is a urate transporter that is primarily expressed in tubules (18), and previous studies have identified that the ABCG2 gene polymorphism causes hyperuricemia and gout (19–21), as well as it revealed the role of renal and extrarenal elimination (22). GLUT9 (SLC2A9, glucose transporter-9) has been identified as urate transporter in mouse renal tubular studied by the same experiment as *Xenopus laevis* oocyte expression system (23). Later, clinical data showed that GLUT9 and URAT1 mutations in human lead to renal hypouricemia involving uric acid reabsorption dysfunction in the proximal tubule (24); meanwhile, benzbromarone and probenecid could inhibit the urate-induced expression of GLUT9 (25). We hypothesized that sunflower calathide extract (SCE) could improve kidney-mediated urate deposition through the UA transporters. It is known that inflammatory cytokines accumulate in the kidney, which results in renal inflammation, and macrophage is a major contributor for renal repair (26) and inflammatory infiltration (27, 28) when the kidney is injured. As usual, RAW264.7 macrophage line, a type of mouse monocytes/macrophages, is commonly used to evaluate the effects of cell proliferation and antiinflammation in LPS-stimulated inflammatory signaling pathway (29, 30). Previous literatures suggested that the COX-2/prostaglandin E2 (PGE2) (31) pathway plays a major role in inflammatory response (32); besides, IFN-γ and NO are important regulatory cytokines on inflammation and LPS-induced activation (33).

Ultrahigh-performance liquid chromatography coupled with mass spectrometry (UPLC-MS/MS) is a rapid and sensitive tool for systematical identification of herbals, and it is widely used to analyze numerous compounds in herbal extracts (34–36). The present study discovered high-matched compounds with a score >80 and screened out bioactive ingredients according to literature reports. To explore the protective mechanism of inflammatory infiltration in the kidney, we investigated the anti-inflammation effect through the classical cellular pathway associated with LPS-induced RAW264.7 cells. Furthermore, we also evaluated the expression levels of URAT1, ABCG2, and GLUT9 in the kidney with the purpose of revealing SCE's possible mechanism on kidney lesions prevention *via* animal experiments *in vivo*. The development of sunflower calathide would reduce agricultural waste, while also encouraging researchers to explore its nutritional and therapeutic properties.

## Materials and Methods

### Plant Materials and Chemicals

Sunflower calathide was obtained from Baicheng city, Jilin Province (12312'45” E, 4452'23” N) in 2019 and identified by professor Shuwen Guan of the School of Life Sciences, Jilin University. The aqueous extract was prepared from dried sunflower calathide powder after passing through a 100-mesh sieve. Fifty grams of powder was added to 600 mL distilled water (1:12, w:v) along with 0.5 g cellulase for enzymolysis. Extraction was carried out at 50°C for 4 h, after which 1 g CaCl_2_ was added to flocculate pectin after filtration, and the solution was subsequently centrifuged at 6,000 rpm for 10 min. The supernatant was spray-dried, yielding 15 g powder, and stored at −80°C. The extract was resuspended in ultrapure water for *in vitro* or *in vivo* experiment administration.

### Sample Processing for UPLC-MS/MS

Around 100 mg SCE powder was added to 1 mL methanol solution (methanol: water, 8:2, V: V), mixed evenly, and centrifuged at 20,000 × g for 10 min in cryogenic centrifuge. The supernatant was filtered through a 0.22-μm PES membrane.

### Identification of SCE by UPLC-ESI-Q-Orbitrap

The sample was subjected to ultrahigh-performance liquid chromatography-ESI-quadrupole-Orbitrap (UPLC-ESI-Q-Orbitrap) to determine its chemical ingredients. A 5 μL-sample of SCE was injected into the column (Thermo Hypersil GOLD 100 × 2.1 mm i.d., 1.9 μm, USA) at a flow rate of 0.300 mL/min and a 5-min gradient at 10°C. A Q-Exactive orbitrap mass spectrometer (Thermo Fisher Scientific Co., Ltd., USA) with an ESI ion source was employed with positive and negative switch modes. The mass spectrometry data of the compounds were analyzed by CD2.1 (Thermo Fisher) and compared with three databases (mzCloud, mzVault, ChemSpider).

### Animals

All animal experimental procedures were approved by the Committee on the Ethics of Animal Experiments of Jilin University of China (Changchun, China), the Animal Use and Care Committee, and the National Institutes of Health guide for the care and use of Laboratory animals (NIH Publications No. 8023, revised 1978). Kunming male mice, weighing 20 ± 2 g, were purchased from Liaoning Changsheng Biotechnology Co., Ltd., China [Certification SCXK (Liao) 2015-0001]. All mice were housed in the Jilin University Experimental Animal Platform and fed a standard diet with free access to sterile water. Animals were maintained under conditions of (22 ± 2)°C, a 12/12 h light/dark cycle, and were allowed to acclimate for 1 week prior to the experiment.

### Hyperuricemia Model and Administration

To induce high UA levels, 84 mice were orally pretreated with yeast extract (Beijing Aoboxing Biotechnology Co., Ltd.) at 20 g/kg once daily for 21 days, with the exception of those intended for the control group (*n* = 12). On day 14 of pretreatment, the mice were divided to 6 groups: model, allopurinol (World Trade Tianjie Pharmaceutical Co., Ltd., Jiangsu, China; 7.6 mg/kg), benzbromarone (Herman Pharmaceuticals, Germany; 7.6 mg/kg), and high-, medium-, and low-dose SCE (2, 1, and 0.5 g/kg, respectively) (*n* = 12 per group). The compound treatment groups were dosed by oral injection once a day for 1 week. The mice were sacrificed 1 h after the last administration on day 21, and the kidneys were taken for H&E staining. Blood was collected from mice with hyperuricemia and centrifuged at 3,000 rpm, 4°C for 15 min to isolate the serum. The levels of UA, xanthine oxidase (XOD), blood urea nitrogen (BUN), and serum creatinine (SCr) in the serum were evaluated using the assay kits (Institute of Bioengineering, Nanjing, CHN), according to the manufacturer's instructions.

### Determination of URAT1, ABCG2, and GLUT9 Transporter Expression in the Kidney

A total of 70 mice were divided into 7 groups: control, model, benzbromarone (7.6 mg/kg), allopurinol (7.6 mg/kg), and low-, medium-, and high-dose SCE (2.5, 5, and 10 g/kg, respectively) (*n* = 10 per group). Following a 15-day oral administration, the kidneys were removed by dissection and stored at −80°C. Subsequently, H&E staining was performed to visualize the structures, and the protein expression levels of the ABCG2, GLUT9, and URAT1 transporters were evaluated by western blotting.

### Cell Culture and Treatment

RAW264.7 cells were purchased from the Cell Bank of the Chinese Academy of Sciences (Shanghai, CHN), and were cultured in DMEM (HyClone, USA) with 10% FBS (ABW, Germany) and 1% penicillin/streptomycin (Solarbio, CHN) at 37°C in humid air containing 5% CO_2_. The cells (3 × 10^4^ cells/well) were seeded in 96-well plates for 24-h cultivation, followed by treating with varying concentrations (2,000; 1,000; 500; 250; 125; and 62.5 μg/mL, respectively), of SCE for 24 h. After 24 h treatment, 10 μl of Cell Counting Kit-8 (CCK-8) reagent (Yeasen, CHN) was added to each well (100 μ*l*) and incubated for 1 h. The absorption values were measured at 450 nm. To evaluate the antiinflammatory effect in LPS-stimulated RAW264.7 cells, the cells were cultured in 24-well (2 × 10^5^ cells/well) and 6-well (9 × 10^5^ cells/well) plates overnight and then pretreated with various doses of SCE (400, 200, 100 μg/ml) for 3 h before LPS stimulated (1 μg/ml, Sigma, USA) for 24 h. Dexamethasone at 10 μM was used as positive control.

ELISA kits were used to evaluate the inflammatory cytokines levels. The cytosolic supernatants were collected after cultured with SCE, and the productions of inflammatory factors NO, PGE2 (R&D Systems, Minneapolis, MN), and IFN-γ (Dakewe Biotech, CHN) were measured by ELISA kits. Western blot was used to detect the regulation of COX-2 in RAW264.7 cells.

### Western Blotting

Kidney tissue was washed three times with sterile PBS to remove residual blood before being sliced into small pieces and homogenized for 60 s in a 10-fold volume of RIPA buffer (Servicebio, CHN). Protease inhibitors were added and the homogenate was incubated on ice for 30 min followed by a 5-min sonication to achieve complete cell lysis. Collected cells were washed three times in PBS and then lysed for 30 min on ice with lysis buffer containing 1% protease inhibitor. The cell lysate was centrifuged at 12,000 rpm, 4°C for 15 min, and the supernatant was collected. Equal amounts of protein were separated on a 5% stacking-10% resolving gel and transferred to polyvinylidene difluoride (PVDF) membrane (Millipore, Bedford, MA). The membranes were blocked in 5% non-fat milk (Servicebio, CHN) with 0.5% TBST (Tris-buffered saline with Tween-20) for 1 h and subsequently incubated overnight at 4°C with gentle rotation with GLUT9 (Novusbio, USA), ABCG2 (Novusbio, USA), and URAT1 (Proteintech Group, USA). After primary antibody incubation, the membranes were washed three times in 1 × TBST (Tris- HCl buffer with 0.5% Tween-20) for 15 min each time, and then incubated with secondary antibody (1:5,000) for 40 min at 37°C. Protein bands were developed with an ECL kit (Solarbio, CHN) and visualized by an automatic imaging system Tanon 5200 after washing three times with secondary antibody.

The lysis and total protein extraction of RAW264.7 cells were performed in the same manner as previously described. The COX-2 primary antibody (Santa Cruz, USA) was used for western blotting.

### H&E Staining and IHC

The removed kidneys were fixed in 4% paraformaldehyde (Solarbio, CHN) solution overnight, followed by paraffin embedding and were sectioned into 4-μm slices. The slices were routinely dewaxed in xylene (Sinopharm Chemical Reagent, CHN), washed in a graded ethanol (Sinopharm Chemical Reagent, CHN) series, and stained with hematoxylin/eosin (HE, Servicebio, CHN). The stained sections were observed under an optical microscope for pathological examination.

After deparaffinizing, rehydrating the paraffin section, followed by antigen retrieval and the elimination of the endogenous peroxidase activity in 3% hydrogen peroxide (Sinopharm Chemical Reagent, CHN), the sections were blocked with 3% BSA (Servicebio, CHN). After the incubation of primary antibody overnight at 4 and the secondary antibody (Servicebio, CHN) for 50 min at room temperature, DAB (Servicebio, CHN) chromogen and hematoxylin counterstaining were used for detection. The sections were dehydrated, sealed, and observed under a microscope.

### Gene Annotation and Analysis

The *M. musculus* Gene ID: ABCG2 (26357), URAT1 (20521), GLUT9 (117591) obtained from NCBI databases (NCBI, Bethesda, MD, USA) was inputted into Metascape (http://metascape.org) and the gene enrichment by network was worked out.

### Statistical Analysis

The values are expressed as the mean ± SEM/SD in multigroup animal experiments. One-way ANOVA was used to analyze the significance of differences, and a value of *p* < 0.05 was considered statistically significant. All statistical analyses were performed using GraphPad Prism version 8.2.1 for Mac (GraphPad Software, La Jolla, CA, USA). IHC and western blot analysis was performed by Image-Pro Plus 6.0 and Alpha Ease FC, which were employed for densitometric analysis.

## Results

### The Major Compound in SCE

**Figure 1:**
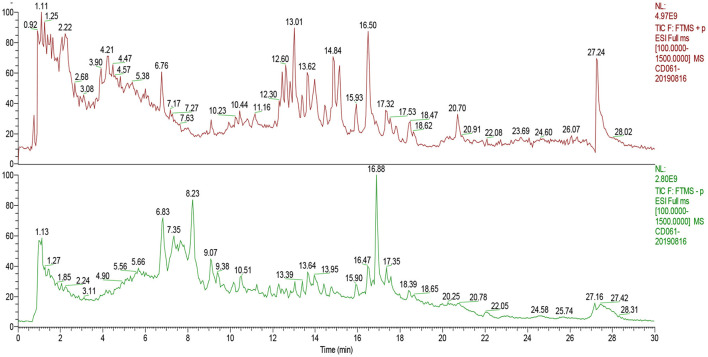
The total ion chromatogram of SCE in positive and negative ion modes.

**Figure 2:**
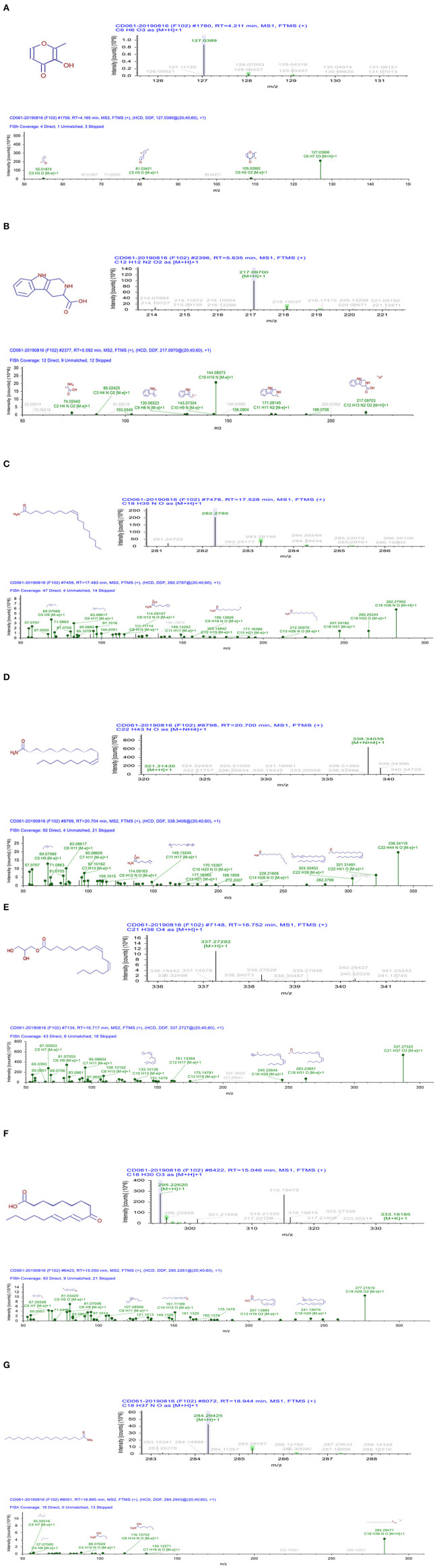
The UPLC-ESI-Q-Orbitrap analysis of SCE (chromatograms and MS1/MS2 spectrum). **(A)** compound 1; **(B)** compound 2; **(C)** compound 3; **(D)** compound 4; **(E)** compound 5; **(F)** compound 6; and **(G)** compound 7.

A total ion chromatogram of SCE in positive and negative ion modes were performed to detect the retention time (Figure 1). The retention time of compound 1 was 4.211 min and the fragment ions C_6_H_7_O_3_ (*m/z* 127.039), C_6_H_5_O_2_ (*m/z* 109.0288), C_5_H_5_O (*m/z* 81.0342), and C_3_H_3_O (*m/z* 55.0187) were observed in MS^2^ (Figure 2A), so that we speculated that compound 1 is maltol. In the positive ion mode, compound 2 (Rt = 5.635) showed an [M+ H]^+^ ion peak at an *m/z* of 217.097 (C_12_H_13_N_2_O_2_). In the MS^2^ spectrum, fragment ions yielded C_10_H_10_N (*m/z* 144.08073) and C_11_H_11_N_2_ (*m/z* 171.09145) according to the MS^1^, indicating that compound 2 is likely to be 2,3,4,9-tetrahydro-1H-β-carboline-3-carboxylic acid (Figure 2B). The retention time of compound 3 was 17.528 min under the same UPLC-MS/MS conditions, and the [M+H]^+^ ion peak was 282.2785, suggesting that its molecular weight may be 281.27–281.28, as well as the molecular formula is predicted to be C_18_H_35_NO due to its fragment ions C_18_H_33_O (*m/z* 265.25244), C_18_H_31_ (*m/z* 247.24182), and C_13_H_26_NO (*m/z* 212.2007) in MS^2^; thus, perhaps compound 3 is oleamide (Figure 2C). Compound 4 (Rt = 20.70 min) showed [M+H]^+^ and [M+NH_4_]^+^ ions at an *m/z* of 321.3143 and 338.34039, indicating that the molecular weight is 320. Also the MS^2^ spectrum showed fragment ions at C_22_H_39_ (*m/z* 303.30453) and C_14_H_28_NO (*m/z* 226.21606), and so compound 4 is most likely to be erucamide (Figure 2D). Further details for compound 5–7 and fragmentation in the mass spectrometry are shown in Figure 2. The compounds present in SCE were deductively characterized by comparing their masses (m/z), fragment ions, and mass spectra with database in Supplementary Material, and the compounds with defined biological activities were selected, whose mzCloud Best Match score was above 80. A total of 8 alkaloids, 5 terpenoids, 10 ketones, 2 flavonoids, 2 glycosides, 3 phenolic, and 2 coumarins compounds are displayed in Table 1.

**Table 1:** Classification of bioactive compounds in sunflower calathide (mzCloud best match score above 80).

**No**.	**Molecular weight**	**Rt(min)**	**Formula**	**Ion mode**	**m/z**	**Identification**	**Category**
1	151.06	4.21	C8 H9 N O2	+	152.0705, 134.0601, 126.0551, 110.0604	Paracetamol	Alkaloid
2	143.09	1.15	C7 H13 N O2	+	144.1019, 98.0969, 84.0814,	DL-Stachydrine	
3	192.04	7.21	C10 H8 O4	+	193.0494, 178.0260, 133.0284	Scopoletin	
4	103.1	1.01	C5 H13 N O	+	104.1074, 60.0817	Choline	
5	168.07	5.99	C11 H8 N2	+	169.0758, 151.0396, 142.0652	Norharman	
6	117.08	1.15	C5 H11 NO2	+	103.0625, 59.0738, 58.0660	Betaine	
7	210.14	6.86	C11 H18 N2 O2	+	183.1494, 138.1277, 114.0916, 98.0605, 86.0970, 70.0659	Cyclo(leucylprolyl)	
8	453.28	27.16	C21 H44 N O7 P	+	452.2782, 171.0052, 152.9946, 146.9600, 96.9681, 78.9575	Glycerophospho-N-palmitoyl ethanolamine	
9	316.2	12.57	C20 H28 O3	+	317.2101, 299.2003, 281.1899, 271.2053, 253.1950	Cafestol	Terpenoid
10	136.13	8.28	C10 H8 O	+	137.1324, 109.0650, 95.0860, 81.0705, 79.0549, 69.0707	Eucalyptol	
11	150.1	9.98	C10 H14 O	+	151.0754, 123.0806, 109.0651, 91.0547, 81.0705, 69.0342	Carvone	
12	152.12	9.05	C10 H16 O	+	135.0442, 109.1015, 107.0859, 93.0704, 81.0705	D- (+)-Camphor	
13	314.19	14.49	C20 H26 O3	+	315.1944, 297.1848, 269.1898, 161.0960, 121.1013	Kahweol	
14	218.17	14.03	C15 H22 O	+	219.1738, 149.0961, 123.1169	Nootkatone	Ketone
15	344.23	13.88	C22 H32 O3	+	345.2415, 327.2319, 299.2363, 281.2263, 241.1948	Medroxyprogesterone	
16	274.19	12.86	C18 H26 O2	+	275.1999, 239.1793, 161.1325, 147.1169, 133.1013	19-Nortestosterone	
17	270.2	13.66	C19 H28 O2	+	271.2049, 253.1949, 225.1636, 197.1325, 145.1012, 121.1014	Dehydroepiandrosterone (DHEA)	
18	302.19	10.97	C19 H26 O3	+	303.1946, 285.1852, 257.1898, 239.1792	11-Ketotestosterone	
19	288.21	11.95	C19 H28 O2	+	289.2154, 271.2052, 253.1949, 243.2106, 187.1482, 161.1329	Testosterone	
20	272.21	14.73	C19 H30 O2	+	255.2104, 227.1790, 199.1480, 145.1010, 131.0855, 105.0702	Etiocholanolone	
21	126.03	4.21	C6 H6 O3	+	127.0390, 109.0288, 81.0342	Maltol	
22	236.1	8.37	C13 H16 O4	+	237.1117, 219.1008, 165.0545, 149.0597, 123.0442, 107.0494	1-[3-Hydroxy-2-(2-hydroxy-2-propanyl)-2,3 -dihydro-1-benzofuran-5-yl] ethanone	
23	272.21	10.7	C19 H30 O2	+	273.2203, 255.2105, 227.1792, 199.1478, 173.1325, 159.1169	Androsterone	
24	374.09	11.57	C19 H18 O8	+	375.1069, 345.0601, 327.0495, 302.0421, 215.0182, 169.0131	5,2'-Dihydroxy-6,7,8,6'-tetramethoxyflavone	Flavonoid
25	314.08	8.79	C17 H14 O6	+	315.0859, 299.0549, 282.0522, 254.0571, 226.0624	Scrophulein	
26	364.09	1.09	C12 H22 O11	+	365.1053, 203.0525, 185.0421, 118.0864	D- (+)-Maltose	Glycosides
27	568.22	7.31	C26 H34 O11	-	567.2082, 359.1502, 329.1393, 175.0756, 160.0519	Lariciresinol 4-O-glucoside	
28	354.1	5.59	C16 H18 O9	-	353.0880, 191.0553, 179.0341, 173.0446, 135.0439	Chlorogenic acid	Phenolic
29	354.1	4.62	C16 H18 O9	-	353.0880, 191.0553, 179.0341, 173.0446, 135.0439	Neochlorogenic acid	
30	126.03	2.26	C6 H6 O3	+	127.0390, 109.0288, 99.0446,	Phloroglucinol	
31	192.04	6.18	C10 H8 O4	+	193.0495, 165.0546, 137.0597, 109.0288, 81.0705	5,7-Dihydroxy-4-methylcoumarin	Coumarins
32	206.06	10.44	C11 H10 O4	+	207.0650, 192.0417,164.0467, 151.0752, 121.0650	Scoparone	

### Body Weight

**Figure 3:**
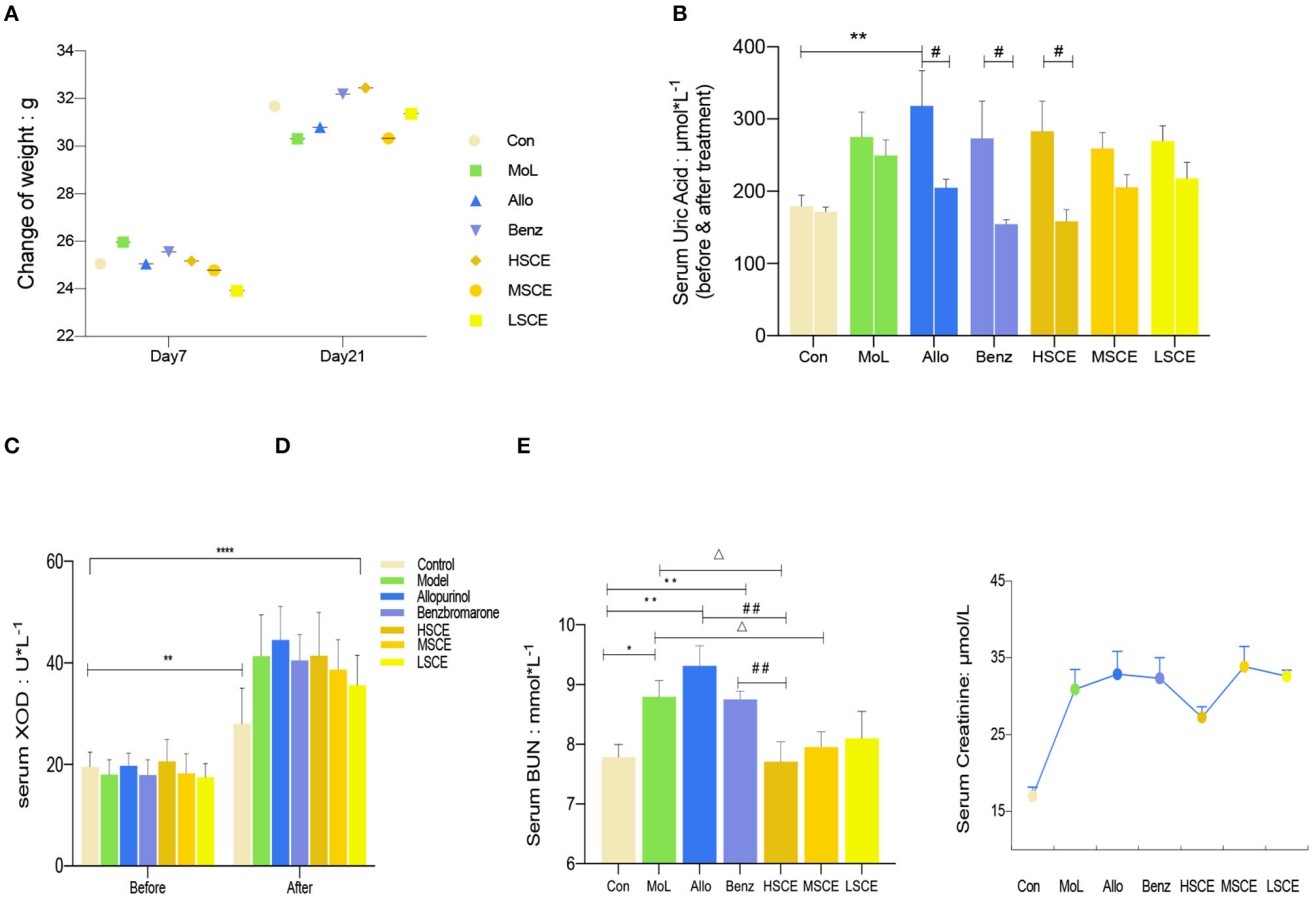
**(A)** Body weight during induction vs. after treatment. **(B)** Serum UA levels, after induction vs. after treatment. **p < 0.01 vs. control, #*p* < 0.05 vs. the same dosage. **(C)** XOD level, before induction vs. after treatment. **p < 0.01 vs. control, ****p < 0.0001 vs. the same dosage. **(D)** Serum BUN levels after treatment with different dosages. *p < 0.05, **p < 0.01 vs. control; ##*p* < 0.01 vs. HSCE; Δ*p* < 0.05 vs. model. Values are presented as the mean ± SEM. Statistical significance is shown above the bar. **(E)** Serum creatinine levels expressed as the mean with SEM. Statistical analyses were performed by one-way ANOVA followed by Bartlett's test; *p* < 0.05 vs control group in all the groups.

All mice gained weight after the 21-day experimental period, with the highest increase in the HSCE group (25.17 ± 0.47, 32.45 ± 0.84 g) and the least increase in the model group (25.96 ± 0.41, 30.31 ± 0.86 g). No statistically significant differences in body weight were observed among the groups during the experiment (Figure 3A).

### Factors Related to Hyperuricemia Model

The UA levels in all groups increased after the 14-day induction with yeast extract as compared with that in the control group (179.33 ± 15.66 μmol/L), with the maximum increase seen in the allopurinol group (318.26 ± 48.92 μmol/L, *p* < 0.01). However, there were no significant changes in the control and model groups with respect to serum UA levels of themselves during the experiment. Treatment with allopurinol (318.26 ± 48.92 μmol/L, 204.81 ± 11.63 μmol/L, *p* < 0.05), benzbromarone (273.05 ± 51.63 μmol/L, 154.5 ± 6.15 μmol/L, *p* < 0.05), and HSCE (283.09 ± 41.89 μmol/L, 158.62 ± 16.29 μmol/L, *p* < 0.05) significantly reduced the UA levels (Figure 3B). The XOD level increased markedly in each group following administration as compared with the initial value (*p* < 0.0001), apart from the control group (*p* = 0.0032) (Figure 3C). Moreover, the XOD level increased in a dose-dependent manner following administration of SCE.

### Biomarkers Involved in Kidney

The serum levels of BUN increased following treatment, except in the HSCE group (7.71 ± 0.332 mmol/L). In comparison with the control group, the serum levels of BUN in the allopurinol (9.331 ± 0.337 mmol/L), benzbromarone (8.75 ± 0.139 mmol/L), and model (8.794 ± 0.275 mmol/L) groups increased over time. The levels in the allopurinol and benzbromarone groups were higher than that in the HSCE (7.71 ± 0.332 mmol/L) group. In comparison with the model group, the serum BUN levels following treatment with SCE decreased in a dose-dependent manner; however, the difference was not significant in the LSCE (8.098 ± 0.451 mmol/L) group (Figure 3D). The serum SCr levels were increased in all the groups following treatment, with the HSCE group having the least increase (Figure 3E). The values were significantly correlated according to Bartlett's test (*p* < 0.05).

### Effect of SCE on the Expression of Renal Transporters

**Figure 4:**
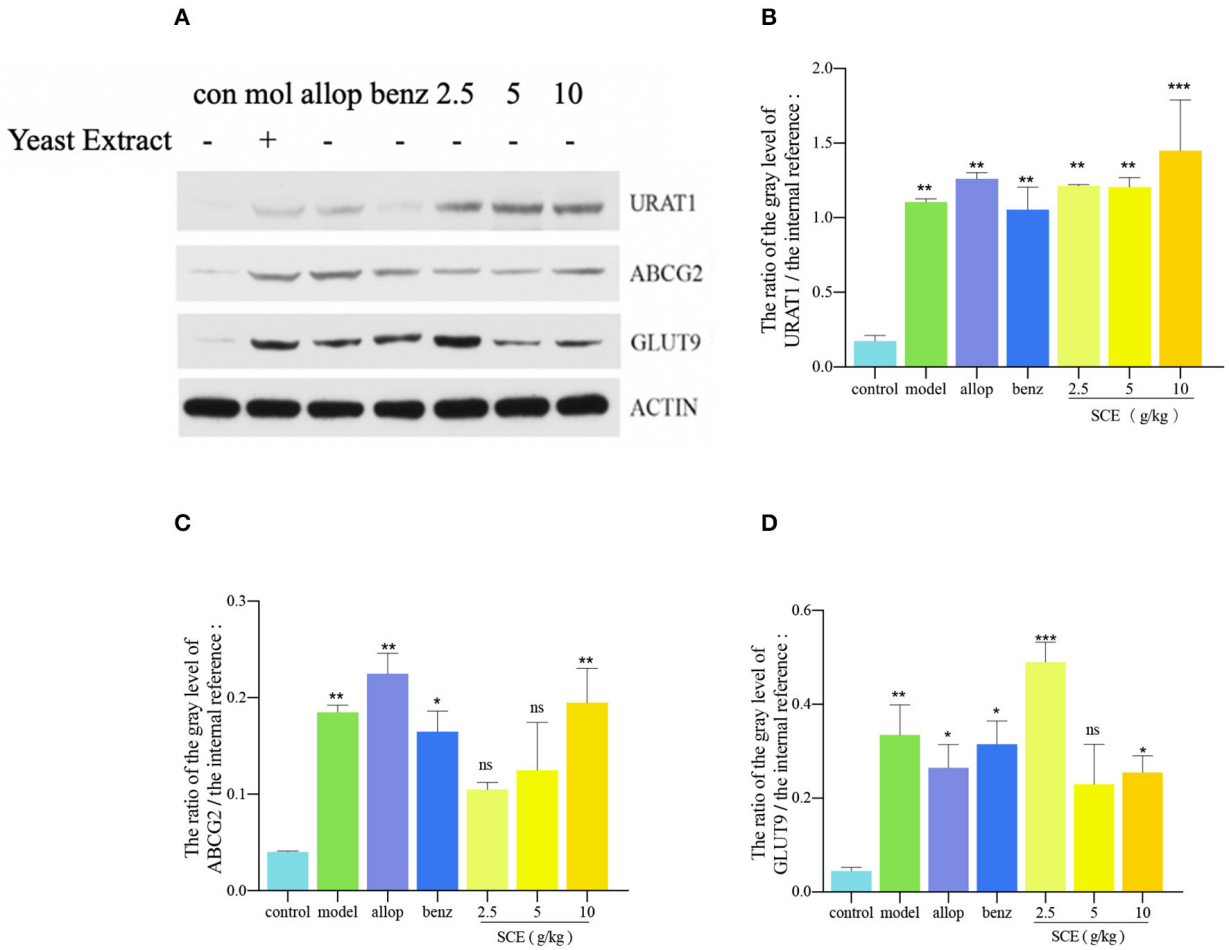
**(A)** The levels of **(B)** URAT1, **(C)** ABCG2, and **(D)** GLUT9 transporter expression in kidney tissue after a 15-day administration. Only the model group was treated with yeast extract. Data analyzed by two-way ANOVA. (*p < 0.05, **p < 0.01, ***p < 0.001, compared with the control group). Densiometric value calculated using the Alpha Ease FC software. Data represented as the mean ± SD from three independent experiments.

Following treatment with SCE, the expression level of URAT1 and ABCG2 in healthy kidney were markedly increased by hSCE treatment as compared with that in the control group (^***^p < 0.001, ^**^p < 0.01) and displayed a dose-dependent increase. A little downregulation of URAT1, ABCG2, and GLUT9 existed in the benzbromarone group when compared with the model group. With respect to the expression level of GLUT9, the decrease was seen in the allopurinol, the benzbromarone, the mSCE, and the hSCE group as compared with the model group; however, the lSCE group was upregulated to approximately twice as that in the hSCE (Figure 4).

### Hematoxylin-Eosin (H&E) Staining of Kidney Tissue

**Figure 5:**
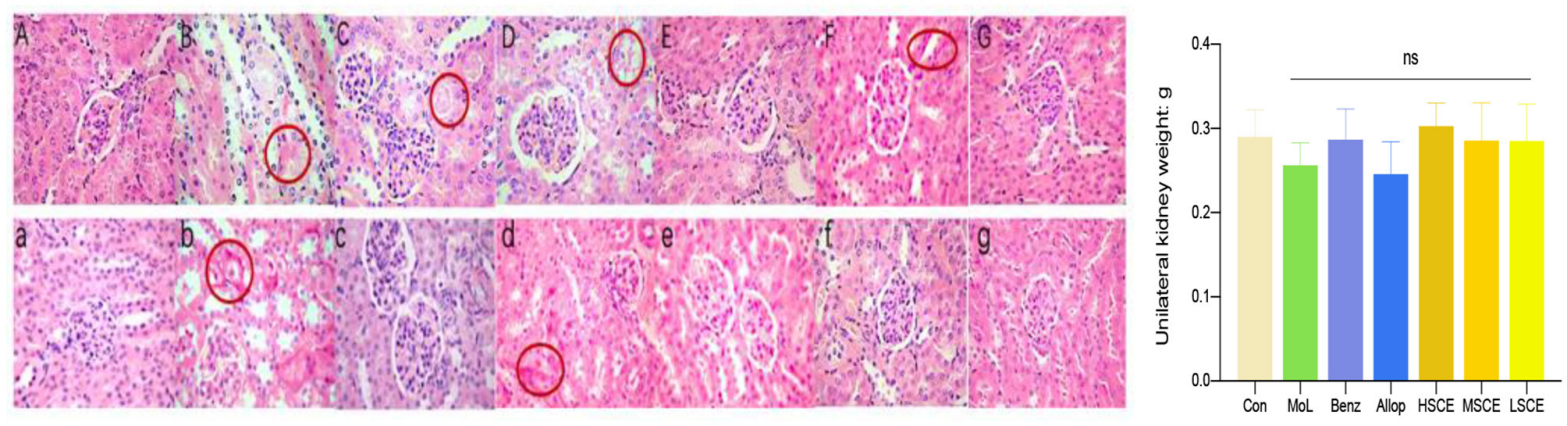
Representative images of renal pathological H&E staining with hyperuricemia **(A–G)** (original magnification, 400×). **(A)** Control group; **(B)** Model group; **(C)** Benzbromarone group (with hyperuricemia); **(D)** Allopurinol group (with hyperuricemia); **(E)** HSCE group; **(F)** MSCE group; **(G)** LSCE group; (a) control group; (b) model group; (c) benzbromarone group; (d) allopurinol group; (e) lSCE group; (f) mSCE group; and (g) hSCE group. Circles indicate area of kidney lesions. The histogram represented the evaluation of unilateral kidney weight and displayed with mean ± SD.

The kidney sections were examined under a microscope (magnification, 400×). The glomeruli in control groups (Figures 5A,a) were plump, the border of the renal capsule was well-defined, and the structure of the renal tubules was normal. There was apparent vacuolization of renal tubules and acidophilic degeneration in the model group (Figures 5B,b), and only the tubular protein cast in the Benzbromarone group (Figure 5C), whereas inflammatory infiltration of renal interstitial cells and renal tubular cell cavitation could be observed in the allopurinol group (Figures 5D,d). There was no obvious vacuolar degeneration or atrophy among the SCE treatment groups, except for the eosinophilic infiltrations in the LSCE group, which might be due to the urate (Figures 5E–G,e–g). The unilateral kidney weight had no significance according to the analysis of the results below.

### Immunohistochemistry

**Figure 6:**
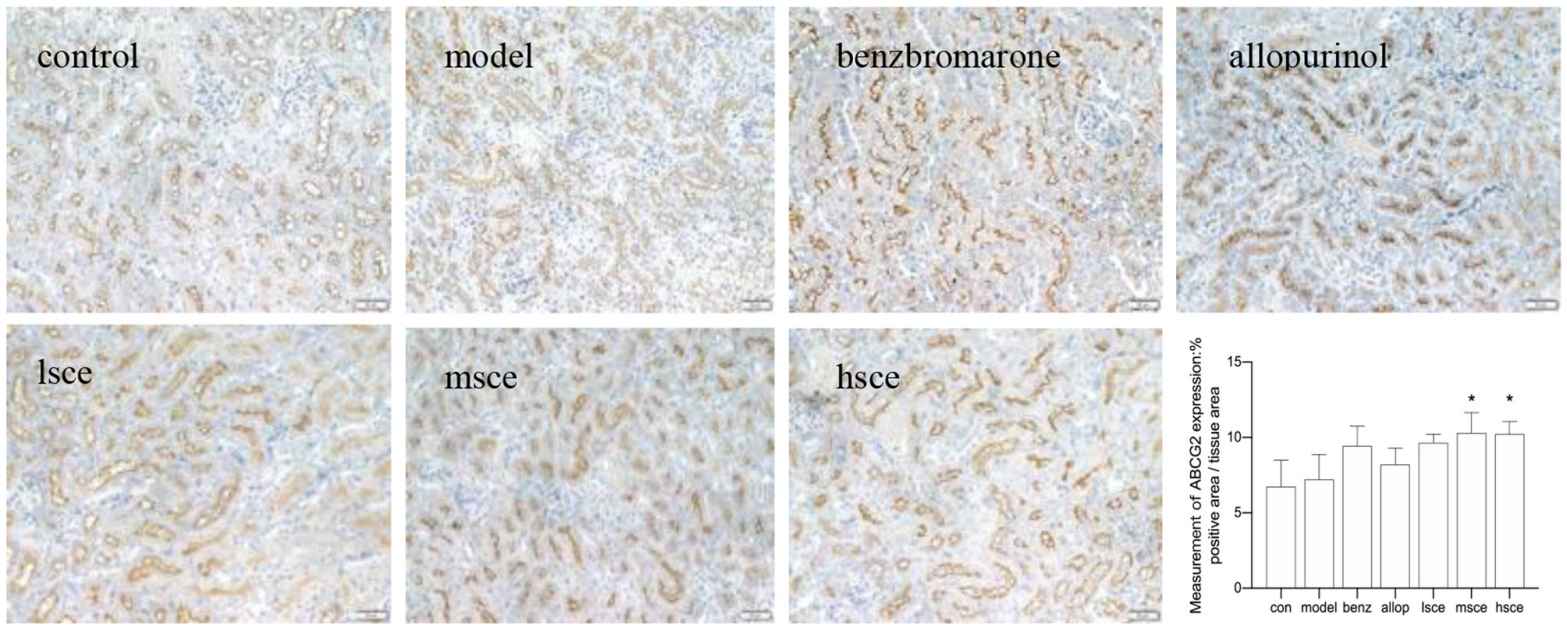
The IHC analysis of ABCG2 expression in kidney after administration (200×). *p < 0.05 vs. control. The value of positive area was analyzed by Image-Pro Plus 6.0. The data were averaged from no less than three different perspectives.

**Figure 7:**
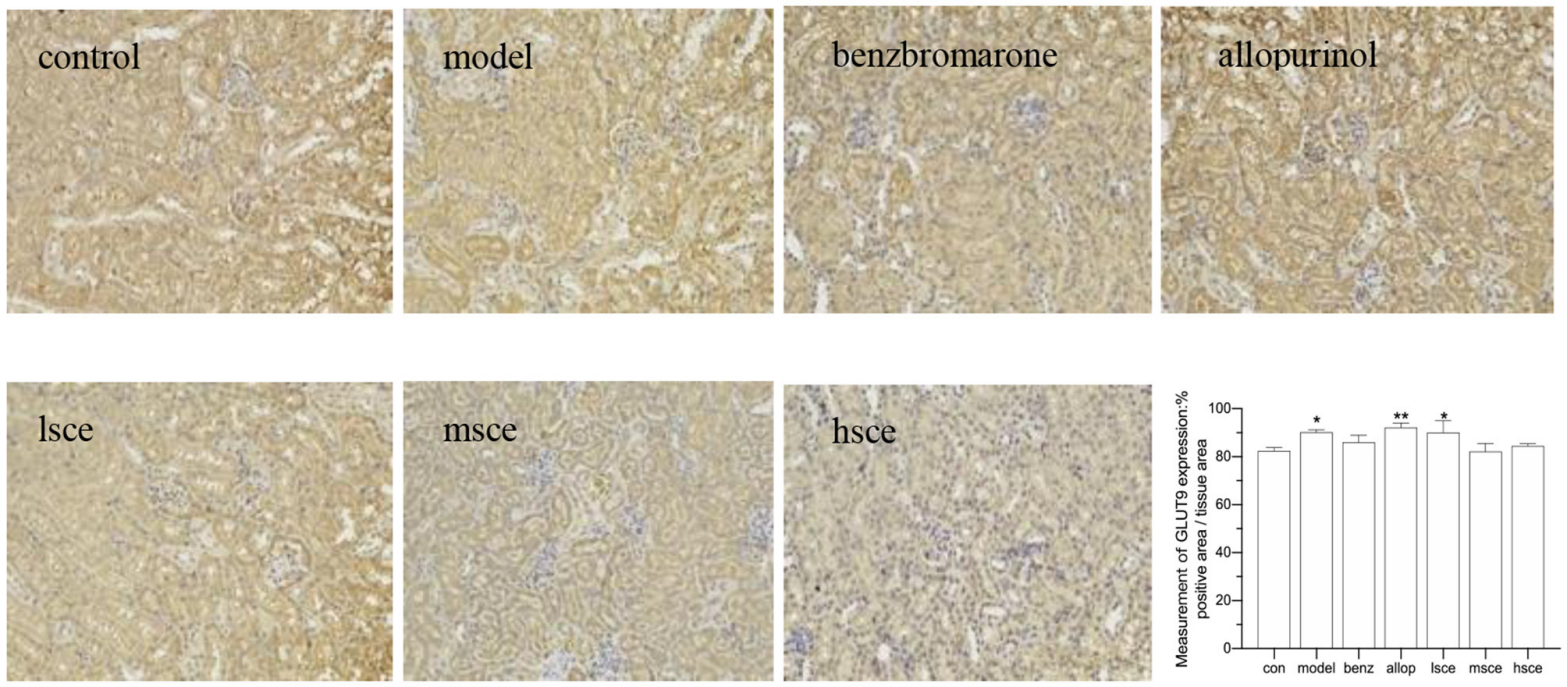
The IHC analysis of GLUT9 expression in kidney after administration (200×). *p < 0.05, **p < 0.01 vs. control. The value of positive area was analyzed by Image-Pro Plus 6.0. The data were averaged from no less than three different perspectives.

**Figure 8:**
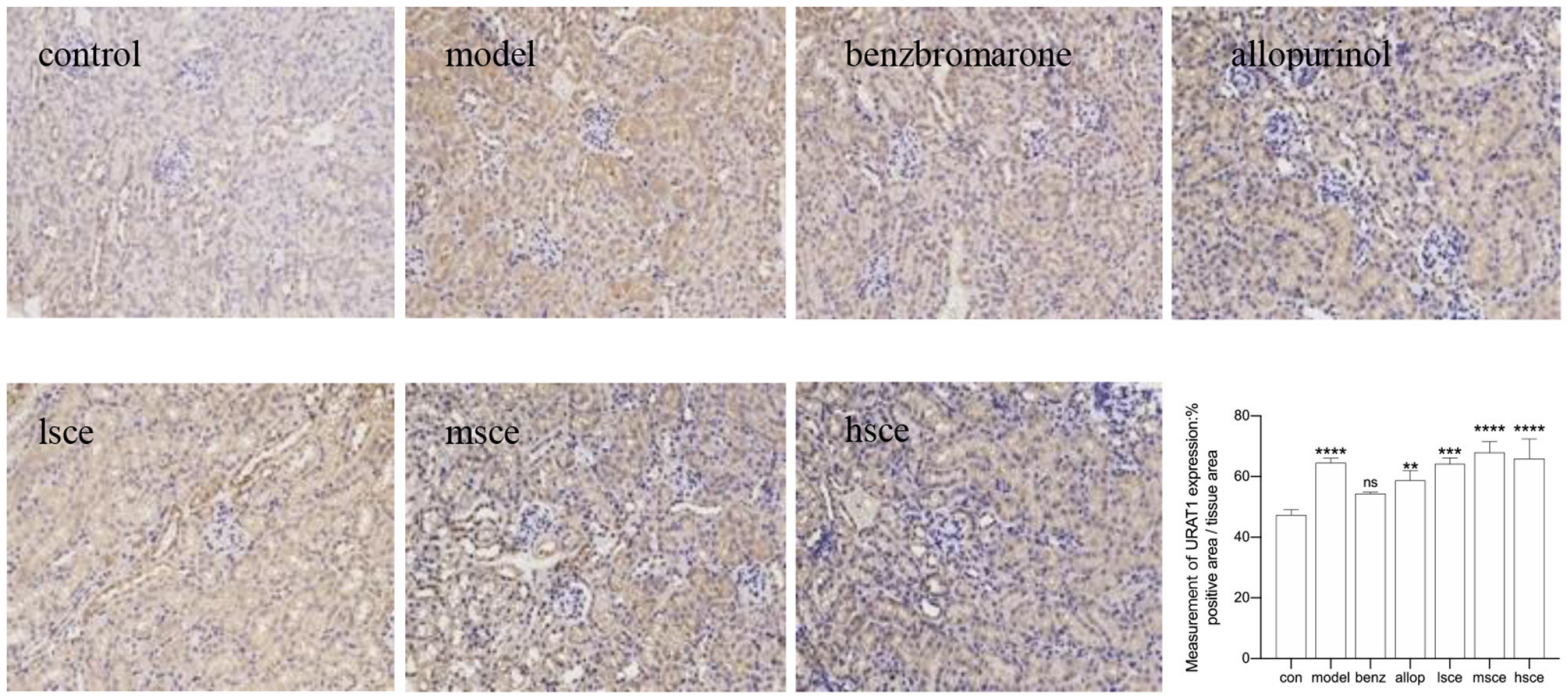
The IHC analysis of URAT1 expression in kidney after administration (200×). **p < 0.01, ***p < 0.001, ****p < 0.0001 vs. control. The value of positive area was analyzed by Image-Pro Plus 6.0. The data were averaged from no less than three different perspectives.

A variable degree of positive reaction was seen in the kidney cortex and the IHC showed wide expression of ABCG2 across the cytoplasm of epithelial cells of tubular in all the groups, particularly the mSCE and hSCE group (^*^P < 0.05), which showed stronger positive reactions compared with the control group (Figure 6). The high magnification images of GLUT9 expression below the microvilli of renal tubules observed ring-like positive reaction in the model (^*^p < 0.05), allopurinol (^**^p < 0.01), and lSCE group (^*^p < 0.05) compared with the control group (Figure 7); meanwhile, the stronger positive localization of URAT1 in the microvilli of tubular cells was not only in the model group (^****^p < 0.0001), but also in the allopurinol (^**^p < 0.01) and SCE (^***^p < *0.001*, ^****^p < *0.0001*) treatment than that of the control group (Figure 8). In the case of kidney cortex and tubular morphology, no kidney lesions were observed.

### Cell Viability and Anti-Inflammatory Activity

**Figure 9:**
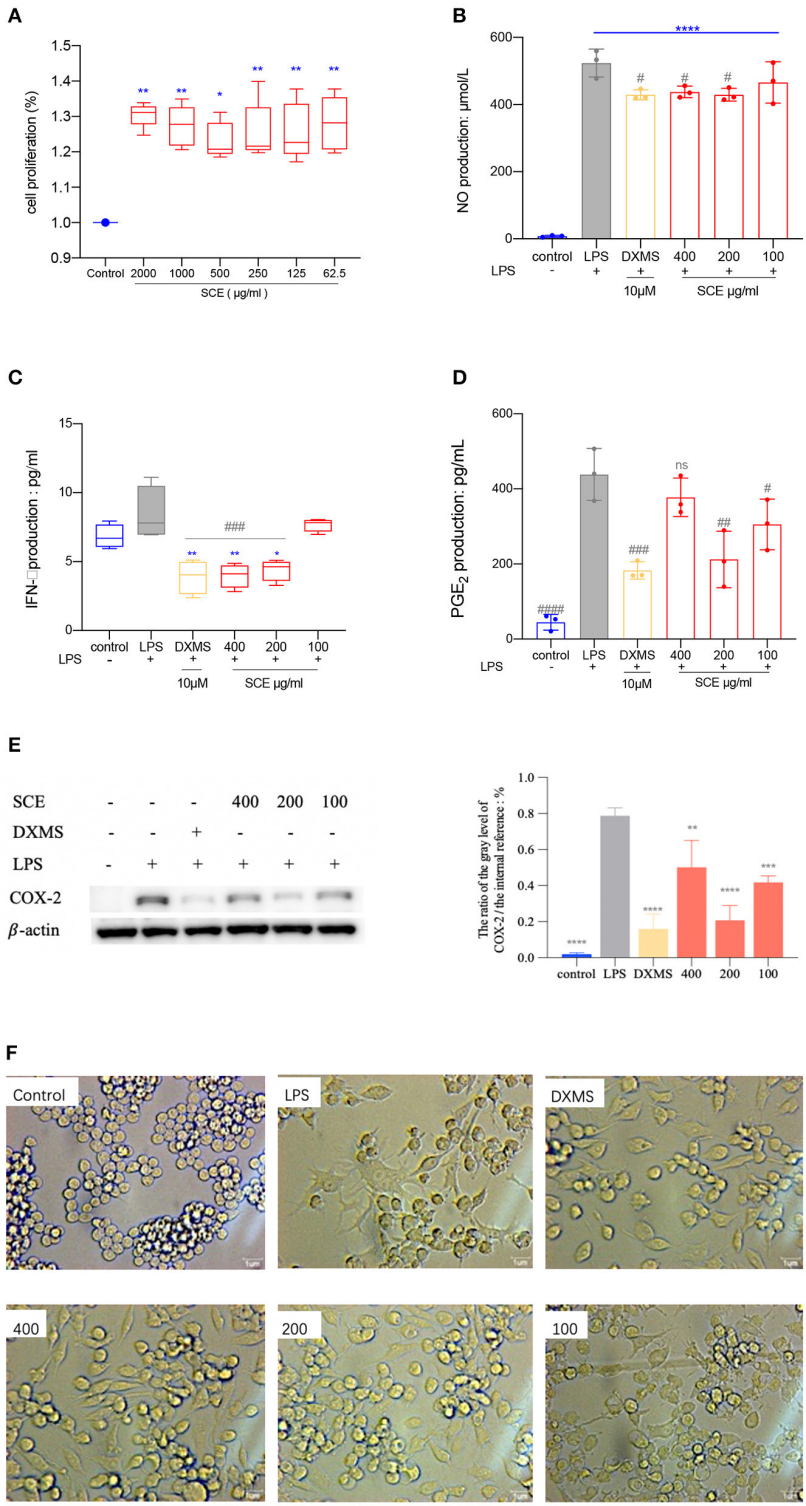
**(A)** The effects of SCE treated with varying concentrations on cell proliferation was detected by CCK-8 assay. The data in control group was set as “1.” Ordinary one-way ANOVA was taken to analyzed multiple comparisons. (*p < 0.05, **p < 0.01, compared with control group). **(B)** The production of NO on LPS-induced RAW264.7 cells (****p < 0.0001 vs. control, #p <0.05 vs. LPS). **(C)** The production of IFN-γ on LPS-induced RAW264.7 cells (*p < 0.05, **p < 0.01 vs. control, ###*p* < 0.001 vs. LPS). **(D)** The production of PGE2 on LPS-induced RAW264.7 cells (#*p* < 0.05, ##*p* < 0.01, ###*p* < 0.001, ####*p* < 0.0001 vs. LPS). **(E)** The expression of COX-2 was evaluated by western blot and analyzed by Image J. (**p < 0.01, ***p < 0.001, ****p < 0.0001 vs. LPS). **(F)** The cell morphology observation after LPS-stimulation and 24-h administration under a bright field microscope (400× magnification). All data were statistically performed by ordinary one-way ANOVA multiple comparisons test and no less than three independent experiments.

RAW264.7 cells proliferation was evaluated after the incubation with different concentrations of SCE: 2,000; 1,000; 500; 250; 125; and 62.5 μg/mL, respectively. The density of formazan product reflects the numbers of live cells in CCK-8 assay. All the concentrations showed enhanced proliferation according to the absorbance at 450 nm when compared with the control group (Figure 9A). The expression levels of NO, IFN-γ, and PGE2 in the LPS-induced RAW264.7 cells were declined in the DXMS (10 μM), 400 μg/ml, and especially 200 μg/ml of SCE treatments (Figures 9B–D). The 200 μg/ml of SCE downregulated COX-2 expression similar to the DXMS treatment as compared with the LPS group (Figure 9E). Under the inverted microscope (400× magnification), the non-treated cells in the control group were spherical and bright, whereas the macrophages in the other groups had numerous pseudopodia and the cultured medium turned yellow, especially the LPS group (Figure 9F).

### Gene Annotation and Analysis of ABCG2, URAT1, and GLUT9

**Figure 10:**
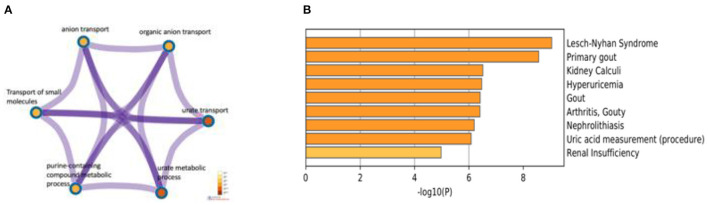
**(A)** Network of enriched terms. Each node represents an enriched term and is colored by its *p*-value. **(B)** Summary of GO enrichment analysis in DisGeMNET^11^, colored by *p*-values.

To identify the relationships between the terms, we used cytoscape tools and came up with six terms relating to urate transport, anion transport, small molecules transport, and purine-containing compound metabolic process (Figure 10A). Gene ontology biological processes, such as kidney calculi, hyperuricemia, nephrolithiasis, uric acid measurement, and renal insufficiency were included, which were involved in this work (Figure 10B).

## Discussion

Due to the fact that the exogenous purines play a vital role in the cause of hyperuricemia (37) and stimulate a high-purine human diet, the hyperuricemia model was established by the intragastric administration of yeast extract. It is acceptable that humans and higher primates lack uricase, but mice possess. The excessive UA can be broken down to allantoin over time in mice (38), thus hyperuricemia model would be induced on a daily basis. Xanthine oxidase (XOD) is an important target for routine treatment of hyperuricemia (39), and inhibiting XOD activity could prevent the conversion of xanthine to UA, so that stronger XOD activity causes higher UA levels. In this work, the serum UA and XOD levels were both markedly elevated following the 14-day yeast extract induction as compared with the control group, indicating that the model was induced successfully. Following treatment, all groups had remarkably decreased serum UA levels as compared with the model group, and the effect of SCE exhibited dose dependency. The levels of BUN and SCr are widely related to the evaluation of renal function in clinical disease (40–42). The allopurinol and benzbromarone groups showed an increase in the levels of serum BUN and SCr, but a significant decline in serum UA levels. However, the HSCE group showed decreased serum UA levels with no elevation in BUN, which is involved in renal lesions (43), and the SCr levels also increased to a lesser extent in the HSCE group. These results indicate that HSCE could reduce the UA levels and prevent the development of urate renal injury.

From the GO analysis, we could draw that ABCG2, URAT1, and GLUT9 transporter proteins play critical roles in hyperuricemia and kidney excretion. Study shows that except for some apical ABC transporters, BCRP/ABCG2 perhaps participates in drug excretion and acts on the kidney (44). As known, the ABCG2 transporter is mainly expressed in the proximal membrane of renal tubular epithelial cells and is solely involved in UA secretion, and the upregulation leads to an improvement in urate excretion (45). Thus, the high expression levels of ABCG2 in the hSCE group suggests a promoting effect on the kidney excretion like benzbromarone and allopurinol, decreasing the UA levels. Recent studies demonstrated that benzbromarone is verified as an URAT1 inhibitor and more effective in human hyperuricemia, as well as preventing the development of CKD as compared with allopurinol (46, 47). Some of them indicated that UA levels negatively regulate URAT1 expression (48, 49); therefore, the result that varying concentrations of SCE upregulated the expression of URAT1 in healthy kidney, which uric acid level is normal, suggest that the SCE perhaps overexpressed URAT1 to maintain the homeostasis of UA. The discovery that SCE inhibits the expression of GLUT9 in normal kidney demonstrated a potential inhibitor. It is well-known that UA levels influence the expression of urate transporters, thus in this work, the mice without hyperuricemia were projected to eliminate the interference of UA. Moreover, the expression of URAT1, ABCG2, and GLUT9 in the model group were all upregulated as compared with the control group according to the above results, validating an influence of UA on the transporters. The H&E staining showed injury and inflammation in the UA-stimulated kidney, indicating that the SCE, at all doses, was effective in ameliorating the kidney injury and phlegmonosis irrespective of whether hyperuricemia developed. Above all, it is reasonable to speculate that SCE may regulate ABCG2 and GLUT9 in a manner similar to benzbromarone, and on account of URAT1 it is abundantly expressed in proximal tubule epithelial cells, the western blot results of URAT1 were slightly different from the IHC even if the IHC of ABCG2, URAT1, and GLUT9 in SCE treatments revealed a similar trend to the western blot. Results from IHC revealed an upregulation of URAT1 and ABCG2 in SCE, whereas the GLUT9 expression levels were lower than any other groups except for the control group in kidney. Besides, the regulation of these transporters, its capacity to accelerate RAW264.7 cells proliferate and attenuate LPS-stimulated inflammation *via* inhibition of the COX-2/PGE2 signaling pathway contributes to macrophages-involved renal repair (50, 51). We found that 200 μg/ml of SCE was the optimal concentration through the inhibitory effects of inflammatory factors.

Due to the limited conditions, four representative compounds that may be of value for further study were preliminarily speculated based on the publications and the UPLC-MS/MS results in the current work. It has been evidenced that Arachidonic acid affects platelets function (52), cell cycle dynamics (53), and renal microvascular function related to its cytochrome P450 metabolites (54, 55). Chlorogenic acid has been reported to have antioxidant and anti-inflammatory properties in the liver and kidney, and studies demonstrate that it is able to attenuate the kidney injury *via* a signaling pathway (56–59). Kahweol has been confirmed to possess anti-inflammatory, anticancer, antitumorigenic, analgesic, etc. properties, as well as a wide range of pharmacological activities (60–62). Scopoletin not only has an antidiabetic effect on enhancing PM- GLUT4 expression in 3T3-L1 adipocytes (63) and has been considered as an AMPK activator for insulin signaling regulation (64), but also has effects on gout by inhibiting urate crystal induced inflammation (65). Moreover, paracetamol, norharman, scoparone, scrophulein, etc. were detected in SCE, all of which have extensive biological and pharmacological properties according to previous studies. We proposed that these ingredients with diverse biological activities have the potential to serve as a material basis for SCE to protect kidney from damage.

This work has certain limitations that more cellular experiments involving URAT1 and GLUT9 expression in renal tubular epithelial cells are expected for further pharmacological research into the mechanisms of hyperuricemia and urate nephrosis. Furthermore, it is necessary to find out the key components acting on the NF-κB signaling pathway and gain insight into the repair mechanism of urate nephropathy. Except for the effect of sunflower calathide in hyperuricemia and nephritic inflammation, other potential pharmaceutical applications are yet to be discovered.

## Conclusion

Taken together, SCE is not only an effective agent that significantly decreased UA levels by regulating the expression of urate transporters URAT1 and ABCG2, especially inhibiting the expression of GLUT9, but it also repaired urate-induced renal injury. To protect the kidney from inflammatory damage, the SCE alleviated the inflammatory response *via* COX-2/PGE2 signaling pathway while also promoting cell proliferation. We speculated some bioactive compounds in SCE perhaps contribute to hyperuricemia. Furthermore, the hyperuricemia model that simulates the human diet, the RAW264.7 cell inflammation model, and the strategic analysis approaches provide precedents for related research. The development of SCE is promising for further nutritional applications.

## Data Availability Statement

The original contributions presented in the study are included in the article/Supplementary Material, further inquiries can be directed to the corresponding author/s.

## Ethics Statement

The animal study was reviewed and approved by Laboratory Animal Ethics Committee, College of Life Sciences, Jilin University.

## Author Contributions

HD: conceptualization, data curation, formal analysis, methodology, project administration, and writing-original draft. ZQ and XZ: resources. SL, KW, CB, and SZ: gavage the animals. XF and WL: funding acquisition, supervision, and review. All authors contributed to the article and approved the submitted version.

## Funding

This study was financially supported by grant from the National Natural Science Foundation of China (No. 31670795).

## Conflict of Interest

The authors declare that the research was conducted in the absence of any commercial or financial relationships that could be construed as a potential conflict of interest.

## Publisher's Note

All claims expressed in this article are solely those of the authors and do not necessarily represent those of their affiliated organizations, or those of the publisher, the editors and the reviewers. Any product that may be evaluated in this article, or claim that may be made by its manufacturer, is not guaranteed or endorsed by the publisher.

## Abbreviations

SCE: sunflower calathide extract
UPLC-ESI-Q-Orbitrap: ultra-high-performance liquid chromatography-ESI-quadrupole-orbitrap
UA: uric acid
BUN: blood urea nitrogen
SCr: serum creatinine
IHC: Immunohistochemistry
HE staining: Haematoxylin-eosin staining
LPS: lipopolysaccharides
DXMS: dexamethasone.
