# Supplementary material for: The Active Components of Sunflower (Helianthus annuus L.) Calathide and the Effects on Urate Nephropathy Based on COX-2/PGE2 Signaling Pathway and the Urate Transporter URAT1, ABCG2, and GLUT9
Source: Front Nutr. 2022 Jan 10;8:769555. doi: 10.3389/fnut.2021.769555 (PMC8784607; doi:10.3389/fnut.2021.769555)
Supplement: Supplementary file 1 [file Data_Sheet_1.docx]

Compounds

19-Aug-2019 3:26

**Structure**

**Name**

**Formula**

**FISh
Coverage**

**RT [min]**

**Molecular Weight**

**mzCloud
Best Match**

**Group Areas**

**Area**

Maltol

C6 H6 O3

80.00

4.21

126.0317

98.6

1

© Reported with Compound Discoverer 2.1

Compounds

19-Aug-2019 3:26

**Structure**

**Name**

**Formula**

**FISh
Coverage**

**RT [min]**

**Molecular Weight**

**mzCloud
Best Match**

**Group Areas**

**Area**

2,3,4,9-
Tetrahydro-1H-β
-carboline-3-
carboxylic acid

C12 H12 N2 O2

59.46

5.63

216.0897

97.0

© Reported with Compound Discoverer 2.1

2

Compounds

19-Aug-2019 3:26

**Structure**

**Name**

**Formula**

**FISh
Coverage**

**RT [min]**

**Molecular Weight**

**mzCloud
Best Match**

**Group Areas**

**Area**

Oleamide

C18 H35 N O

92.63

17.52

281.2713

96.7

© Reported with Compound Discoverer 2.1

3

Compounds

19-Aug-2019 3:26

**Structure**

**Name**

**Formula**

**FISh
Coverage**

**RT [min]**

**Molecular Weight**

**mzCloud
Best Match**

**Group Areas**

**Area**

Oleamide

C18 H35 N O

91.84

17.80

281.2713

96.4

© Reported with Compound Discoverer 2.1

4

Compounds

19-Aug-2019 3:26

**Structure**

**Name**

**Formula**

**FISh
Coverage**

**RT [min]**

**Molecular Weight**

**mzCloud
Best Match**

**Group Areas**

**Area**

Erucamide

C22 H43 N O

93.00

20.71

320.3066

95.9

© Reported with Compound Discoverer 2.1

5

Compounds

19-Aug-2019 3:26

**Structure**

**Name**

**Formula**

**FISh
Coverage**

**RT [min]**

**Molecular Weight**

**mzCloud
Best Match**

**Group Areas**

**Area**

Erucamide

C22 H43 N O

93.33

21.01

337.3331

95.5

© Reported with Compound Discoverer 2.1

6

Compounds

19-Aug-2019 3:26

**Structure**

**Name**

**Formula**

**FISh
Coverage**

**RT [min]**

**Molecular Weight**

**mzCloud
Best Match**

**Group Areas**

**Area**

1-Linoleoyl
glycerol

C21 H38 O4

87.76

16.75

336.2656

95.1

© Reported with Compound Discoverer 2.1

7

Compounds

19-Aug-2019 3:26

**Structure**

**Name**

**Formula**

**FISh
Coverage**

**RT [min]**

**Molecular Weight**

**mzCloud
Best Match**

**Group Areas**

**Area**

9-Oxo-10(E),
12(E)-
octadecadienoic
acid

C18 H30 O3

84.62

15.03

294.2189

94.8

© Reported with Compound Discoverer 2.1

8

Compounds

19-Aug-2019 3:26

**Structure**

**Name**

**Formula**

**FISh
Coverage**

**RT [min]**

**Molecular Weight**

**mzCloud
Best Match**

**Group Areas**

**Area**

Stearamide

C18 H37 N O

100.00

18.93

283.2870

94.1

© Reported with Compound Discoverer 2.1

9

Compounds

19-Aug-2019 3:26

**Structure**

**Name**

**Formula**

**FISh
Coverage**

**RT [min]**

**Molecular Weight**

**mzCloud
Best Match**

**Group Areas**

**Area**

4-
Dodecylbenzen
esulfonic acid

C18 H30 O3 S

25.00

27.11

326.1917

94.0

© Reported with Compound Discoverer 2.1

10

Compounds

19-Aug-2019 3:26

**Structure**

**Name**

**Formula**

**FISh
Coverage**

**RT [min]**

**Molecular Weight**

**mzCloud
Best Match**

**Group Areas**

**Area**

Abietic acid

C20 H30 O2

12.83

302.2235

93.8

© Reported with Compound Discoverer 2.1

11

Compounds

19-Aug-2019 3:26

**Structure**

**Name**

**Formula**

**FISh
Coverage**

**RT [min]**

**Molecular Weight**

**mzCloud
Best Match**

**Group Areas**

**Area**

3-Hydroxy-2-
methylpyridine

C6 H7 N O

1.30

109.0531

93.8

© Reported with Compound Discoverer 2.1

12

Compounds

19-Aug-2019 3:26

**Structure**

**Name**

**Formula**

**FISh
Coverage**

**RT [min]**

**Molecular Weight**

**mzCloud
Best Match**

**Group Areas**

**Area**

α-Eleostearic
acid

C18 H30 O2

16.69

278.2240

93.4

© Reported with Compound Discoverer 2.1

13

Compounds

19-Aug-2019 3:26

**Structure**

**Name**

**Formula**

**FISh
Coverage**

**RT [min]**

**Molecular Weight**

**mzCloud
Best Match**

**Group Areas**

**Area**

9-Oxo-10(E),
12(E)-
octadecadienoic
acid

C18 H30 O3

14.77

294.2190

93.1

© Reported with Compound Discoverer 2.1

14

Compounds

19-Aug-2019 3:26

**Structure**

**Name**

**Formula**

**FISh
Coverage**

**RT [min]**

**Molecular Weight**

**mzCloud
Best Match**

**Group Areas**

**Area**

9S,13R-12-
Oxophytodienoi
c acid

C18 H28 O3

13.77

292.2033

92.9

© Reported with Compound Discoverer 2.1

15

Compounds

19-Aug-2019 3:26

**Structure**

**Name**

**Formula**

**FISh
Coverage**

**RT [min]**

**Molecular Weight**

**mzCloud
Best Match**

**Group Areas**

**Area**

(+/-)12(13)-
DiHOME

C18 H34 O4

14.42

296.2352

92.8

© Reported with Compound Discoverer 2.1

16

Compounds

19-Aug-2019 3:26

**Structure**

**Name**

**Formula**

**FISh
Coverage**

**RT [min]**

**Molecular Weight**

**mzCloud
Best Match**

**Group Areas**

**Area**

α-Eleostearic
acid

C18 H30 O2

14.42

278.2239

92.8

© Reported with Compound Discoverer 2.1

17

Compounds

19-Aug-2019 3:26

**Structure**

**Name**

**Formula**

**FISh
Coverage**

**RT [min]**

**Molecular Weight**

**mzCloud
Best Match**

**Group Areas**

**Area**

Betaine

C5 H11 N O2

1.16

117.0792

92.6

© Reported with Compound Discoverer 2.1

18

Compounds

19-Aug-2019 3:26

**Structure**

**Name**

**Formula**

**FISh
Coverage**

**RT [min]**

**Molecular Weight**

**mzCloud
Best Match**

**Group Areas**

**Area**

Docosanamide

C22 H45 N O

20.18

339.3493

92.4

© Reported with Compound Discoverer 2.1

19

Compounds

19-Aug-2019 3:26

**Structure**

**Name**

**Formula**

**FISh
Coverage**

**RT [min]**

**Molecular Weight**

**mzCloud
Best Match**

**Group Areas**

**Area**

1-Linoleoyl
glycerol

C21 H38 O4

16.74

354.2761

92.4

© Reported with Compound Discoverer 2.1

20

Compounds

19-Aug-2019 3:26

**Structure**

**Name**

**Formula**

**FISh
Coverage**

**RT [min]**

**Molecular Weight**

**mzCloud
Best Match**

**Group Areas**

**Area**

Abietic acid

C20 H30 O2

16.91

302.2246

92.3

© Reported with Compound Discoverer 2.1

21

Compounds

19-Aug-2019 3:26

**Structure**

**Name**

**Formula**

**FISh
Coverage**

**RT [min]**

**Molecular Weight**

**mzCloud
Best Match**

**Group Areas**

**Area**

16-
Hydroxyhexade
canoic acid

C16 H32 O3

17.11

272.2352

92.3

© Reported with Compound Discoverer 2.1

22

Compounds

19-Aug-2019 3:26

**Structure**

**Name**

**Formula**

**FISh
Coverage**

**RT [min]**

**Molecular Weight**

**mzCloud
Best Match**

**Group Areas**

**Area**

2-(8-Hydroxy-
4a,8-
dimethyldecahy
dro-2-
naphthalenyl)
acrylic acid

C15 H24 O3

11.61

234.1611

92.3

© Reported with Compound Discoverer 2.1

23

Compounds

19-Aug-2019 3:26

**Structure**

**Name**

**Formula**

**FISh
Coverage**

**RT [min]**

**Molecular Weight**

**mzCloud
Best Match**

**Group Areas**

**Area**

(3aR,8R,8aR,
9aR)-8-
Hydroxy-8a-
methyl-3,5-
bis(methylene)
decahydronapht
ho[2,3-b]furan-
2(3H)-one

C15 H20 O3

10.31

248.1405

92.3

© Reported with Compound Discoverer 2.1

24

Compounds

19-Aug-2019 3:26

**Structure**

**Name**

**Formula**

**FISh
Coverage**

**RT [min]**

**Molecular Weight**

**mzCloud
Best Match**

**Group Areas**

**Area**

Ageratriol

C15 H24 O3

10.51

234.1611

91.9

© Reported with Compound Discoverer 2.1

25

Compounds

19-Aug-2019 3:26

**Structure**

**Name**

**Formula**

**FISh
Coverage**

**RT [min]**

**Molecular Weight**

**mzCloud
Best Match**

**Group Areas**

**Area**

α-Eleostearic
acid

C18 H30 O2

17.12

278.2239

91.9

© Reported with Compound Discoverer 2.1

26

Compounds

19-Aug-2019 3:26

**Structure**

**Name**

**Formula**

**FISh
Coverage**

**RT [min]**

**Molecular Weight**

**mzCloud
Best Match**

**Group Areas**

**Area**

α-Eleostearic
acid

C18 H30 O2

12.92

278.2239

91.9

© Reported with Compound Discoverer 2.1

27

Compounds

19-Aug-2019 3:26

**Structure**

**Name**

**Formula**

**FISh
Coverage**

**RT [min]**

**Molecular Weight**

**mzCloud
Best Match**

**Group Areas**

**Area**

Carvone

C10 H14 O

9.98

150.1041

91.9

© Reported with Compound Discoverer 2.1

28

Compounds

19-Aug-2019 3:26

**Structure**

**Name**

**Formula**

**FISh
Coverage**

**RT [min]**

**Molecular Weight**

**mzCloud
Best Match**

**Group Areas**

**Area**

Hexadecanamid
e

C16 H33 N O

17.29

255.2558

91.8

© Reported with Compound Discoverer 2.1

29

Compounds

19-Aug-2019 3:26

**Structure**

**Name**

**Formula**

**FISh
Coverage**

**RT [min]**

**Molecular Weight**

**mzCloud
Best Match**

**Group Areas**

**Area**

9S,13R-12-
Oxophytodienoi
c acid

C18 H28 O3

16.35

292.2032

91.8

© Reported with Compound Discoverer 2.1

30

Compounds

19-Aug-2019 3:26

**Structure**

**Name**

**Formula**

**FISh
Coverage**

**RT [min]**

**Molecular Weight**

**mzCloud
Best Match**

**Group Areas**

**Area**

Nootkatone

C15 H22 O

14.04

218.1665

91.4

© Reported with Compound Discoverer 2.1

31

Compounds

19-Aug-2019 3:26

**Structure**

**Name**

**Formula**

**FISh
Coverage**

**RT [min]**

**Molecular Weight**

**mzCloud
Best Match**

**Group Areas**

**Area**

Bis(4-
ethylbenzyliden
e)sorbitol

C24 H30 O6

12.81

414.2028

91.3

© Reported with Compound Discoverer 2.1

32

Compounds

19-Aug-2019 3:26

**Structure**

**Name**

**Formula**

**FISh
Coverage**

**RT [min]**

**Molecular Weight**

**mzCloud
Best Match**

**Group Areas**

**Area**

Betaine

C5 H11 N O2

1.53

117.0792

91.1

© Reported with Compound Discoverer 2.1

33

Compounds

19-Aug-2019 3:26

**Structure**

**Name**

**Formula**

**FISh
Coverage**

**RT [min]**

**Molecular Weight**

**mzCloud
Best Match**

**Group Areas**

**Area**

Arachidonic acid

C20 H32 O2

17.45

286.2293

91.1

© Reported with Compound Discoverer 2.1

34

Compounds

19-Aug-2019 3:26

**Structure**

**Name**

**Formula**

**FISh
Coverage**

**RT [min]**

**Molecular Weight**

**mzCloud
Best Match**

**Group Areas**

**Area**

16-
Hydroxyhexade
canoic acid

C16 H32 O3

16.87

272.2352

90.9

© Reported with Compound Discoverer 2.1

35

Compounds

19-Aug-2019 3:26

**Structure**

**Name**

**Formula**

**FISh
Coverage**

**RT [min]**

**Molecular Weight**

**mzCloud
Best Match**

**Group Areas**

**Area**

7-Methyl-3-
methylene-6-(3-
oxobutyl)-
3,3a,4,7,8,8a-
hexahydro-2H-
cyclohepta[b]
furan-2-one

C15 H20 O3

10.29

230.1299

90.9

© Reported with Compound Discoverer 2.1

36

Compounds

19-Aug-2019 3:26

**Structure**

**Name**

**Formula**

**FISh
Coverage**

**RT [min]**

**Molecular Weight**

**mzCloud
Best Match**

**Group Areas**

**Area**

9-Oxo-10(E),
12(E)-
octadecadienoic
acid

C18 H30 O3

10.51

294.2189

90.9

© Reported with Compound Discoverer 2.1

37

Compounds

19-Aug-2019 3:26

**Structure**

**Name**

**Formula**

**FISh
Coverage**

**RT [min]**

**Molecular Weight**

**mzCloud
Best Match**

**Group Areas**

**Area**

Myristyl sulfate

C14 H30 O4 S

24.61

294.1867

90.8

© Reported with Compound Discoverer 2.1

38

Compounds

19-Aug-2019 3:26

**Structure**

**Name**

**Formula**

**FISh
Coverage**

**RT [min]**

**Molecular Weight**

**mzCloud
Best Match**

**Group Areas**

**Area**

Ageratriol

C15 H24 O3

13.88

234.1611

90.7

© Reported with Compound Discoverer 2.1

39

Compounds

19-Aug-2019 3:26

**Structure**

**Name**

**Formula**

**FISh
Coverage**

**RT [min]**

**Molecular Weight**

**mzCloud
Best Match**

**Group Areas**

**Area**

Etiocholanolone

C19 H30 O2

14.73

272.2135

90.5

© Reported with Compound Discoverer 2.1

40

Compounds

19-Aug-2019 3:26

**Structure**

**Name**

**Formula**

**FISh
Coverage**

**RT [min]**

**Molecular Weight**

**mzCloud
Best Match**

**Group Areas**

**Area**

2-Isopropylmalic
acid

C7 H12 O5

5.25

176.0677

90.2

© Reported with Compound Discoverer 2.1

41

Compounds

19-Aug-2019 3:26

**Structure**

**Name**

**Formula**

**FISh
Coverage**

**RT [min]**

**Molecular Weight**

**mzCloud
Best Match**

**Group Areas**

**Area**

Lariciresinol 4-
O-glucoside

C26 H34 O11

7.31

568.2155

90.1

© Reported with Compound Discoverer 2.1

42

Compounds

19-Aug-2019 3:26

**Structure**

**Name**

**Formula**

**FISh
Coverage**

**RT [min]**

**Molecular Weight**

**mzCloud
Best Match**

**Group Areas**

**Area**

Adenine

C5 H5 N5

1.19

135.0545

90.0

© Reported with Compound Discoverer 2.1

43

Compounds

19-Aug-2019 3:26

**Structure**

**Name**

**Formula**

**FISh
Coverage**

**RT [min]**

**Molecular Weight**

**mzCloud
Best Match**

**Group Areas**

**Area**

Nicotinic acid

C6 H5 N O2

1.25

123.0322

89.8

© Reported with Compound Discoverer 2.1

44

Compounds

19-Aug-2019 3:26

**Structure**

**Name**

**Formula**

**FISh
Coverage**

**RT [min]**

**Molecular Weight**

**mzCloud
Best Match**

**Group Areas**

**Area**

Cafestol

C20 H28 O3

12.57

316.2028

89.8

© Reported with Compound Discoverer 2.1

45

Compounds

19-Aug-2019 3:26

**Structure**

**Name**

**Formula**

**FISh
Coverage**

**RT [min]**

**Molecular Weight**

**mzCloud
Best Match**

**Group Areas**

**Area**

Scopoletin

C10 H8 O4

7.21

192.0419

89.7

© Reported with Compound Discoverer 2.1

46

Compounds

19-Aug-2019 3:26

**Structure**

**Name**

**Formula**

**FISh
Coverage**

**RT [min]**

**Molecular Weight**

**mzCloud
Best Match**

**Group Areas**

**Area**

19-
Nortestosterone

C18 H26 O2

12.85

274.1925

89.7

© Reported with Compound Discoverer 2.1

47

Compounds

19-Aug-2019 3:26

**Structure**

**Name**

**Formula**

**FISh
Coverage**

**RT [min]**

**Molecular Weight**

**mzCloud
Best Match**

**Group Areas**

**Area**

Kahweol

C20 H26 O3

13.03

314.1871

89.7

© Reported with Compound Discoverer 2.1

48

Compounds

19-Aug-2019 3:26

**Structure**

**Name**

**Formula**

**FISh
Coverage**

**RT [min]**

**Molecular Weight**

**mzCloud
Best Match**

**Group Areas**

**Area**

Kahweol

C20 H26 O3

14.48

314.1871

89.6

© Reported with Compound Discoverer 2.1

49

Compounds

19-Aug-2019 3:26

**Structure**

**Name**

**Formula**

**FISh
Coverage**

**RT [min]**

**Molecular Weight**

**mzCloud
Best Match**

**Group Areas**

**Area**

1-Linoleoyl
glycerol

C21 H38 O4

16.55

354.2761

89.6

© Reported with Compound Discoverer 2.1

50

Compounds

19-Aug-2019 3:26

**Structure**

**Name**

**Formula**

**FISh
Coverage**

**RT [min]**

**Molecular Weight**

**mzCloud
Best Match**

**Group Areas**

**Area**

1,4a-Dimethyl-
6-methylene-5-
[2-(2-oxo-2,5-
dihydro-3-
furanyl)ethyl]
decahydro-1-
naphthalenecar
boxylic acid

C20 H28 O4

12.41

332.1976

89.5

© Reported with Compound Discoverer 2.1

51

Compounds

19-Aug-2019 3:26

**Structure**

**Name**

**Formula**

**FISh
Coverage**

**RT [min]**

**Molecular Weight**

**mzCloud
Best Match**

**Group Areas**

**Area**

Ageratriol

C15 H24 O3

11.86

234.1612

89.4

© Reported with Compound Discoverer 2.1

52

Compounds

19-Aug-2019 3:26

**Structure**

**Name**

**Formula**

**FISh
Coverage**

**RT [min]**

**Molecular Weight**

**mzCloud
Best Match**

**Group Areas**

**Area**

1,4a-Dimethyl-
6-methylene-5-
[2-(2-oxo-2,5-
dihydro-3-
furanyl)ethyl]
decahydro-1-
naphthalenecar
boxylic acid

C20 H28 O4

12.13

332.1976

89.2

© Reported with Compound Discoverer 2.1

53

Compounds

19-Aug-2019 3:26

**Structure**

**Name**

**Formula**

**FISh
Coverage**

**RT [min]**

**Molecular Weight**

**mzCloud
Best Match**

**Group Areas**

**Area**

Kahweol

C20 H26 O3

15.17

314.1871

89.2

© Reported with Compound Discoverer 2.1

54

Compounds

19-Aug-2019 3:26

**Structure**

**Name**

**Formula**

**FISh
Coverage**

**RT [min]**

**Molecular Weight**

**mzCloud
Best Match**

**Group Areas**

**Area**

7,8-
Bis
(hydroxymethyl)
-1,4a-dimethyl-
3,4,4a,5,6,7-
hexahydro-2H-
benzo[7]
annulen-2-one

C15 H22 O3

10.79

250.1561

89.1

© Reported with Compound Discoverer 2.1

55

Compounds

19-Aug-2019 3:26

**Structure**

**Name**

**Formula**

**FISh
Coverage**

**RT [min]**

**Molecular Weight**

**mzCloud
Best Match**

**Group Areas**

**Area**

D-(+)-Camphor

C10 H16 O

9.05

152.1197

88.9

© Reported with Compound Discoverer 2.1

56

Compounds

19-Aug-2019 3:26

**Structure**

**Name**

**Formula**

**FISh
Coverage**

**RT [min]**

**Molecular Weight**

**mzCloud
Best Match**

**Group Areas**

**Area**

Docosanamide

C22 H45 N O

22.23

339.3493

88.7

© Reported with Compound Discoverer 2.1

57

Compounds

19-Aug-2019 3:26

**Structure**

**Name**

**Formula**

**FISh
Coverage**

**RT [min]**

**Molecular Weight**

**mzCloud
Best Match**

**Group Areas**

**Area**

Abietic acid

C20 H30 O2

17.36

302.2246

88.7

© Reported with Compound Discoverer 2.1

58

Compounds

19-Aug-2019 3:26

**Structure**

**Name**

**Formula**

**FISh
Coverage**

**RT [min]**

**Molecular Weight**

**mzCloud
Best Match**

**Group Areas**

**Area**

19-
Nortestosterone

C18 H26 O2

10.17

274.1925

88.6

© Reported with Compound Discoverer 2.1

59

Compounds

19-Aug-2019 3:26

**Structure**

**Name**

**Formula**

**FISh
Coverage**

**RT [min]**

**Molecular Weight**

**mzCloud
Best Match**

**Group Areas**

**Area**

Stearic acid

C18 H36 O2

19.96

284.2718

88.4

© Reported with Compound Discoverer 2.1

60

Compounds

19-Aug-2019 3:26

**Structure**

**Name**

**Formula**

**FISh
Coverage**

**RT [min]**

**Molecular Weight**

**mzCloud
Best Match**

**Group Areas**

**Area**

(+/-)12(13)-
DiHOME

C18 H34 O4

15.47

296.2343

88.4

© Reported with Compound Discoverer 2.1

61

Compounds

19-Aug-2019 3:26

**Structure**

**Name**

**Formula**

**FISh
Coverage**

**RT [min]**

**Molecular Weight**

**mzCloud
Best Match**

**Group Areas**

**Area**

Choline

C5 H13 N O

1.00

103.1001

88.3

© Reported with Compound Discoverer 2.1

62

Compounds

19-Aug-2019 3:26

**Structure**

**Name**

**Formula**

**FISh
Coverage**

**RT [min]**

**Molecular Weight**

**mzCloud
Best Match**

**Group Areas**

**Area**

1,4a-Dimethyl-
6-methylene-5-
[2-(2-oxo-2,5-
dihydro-3-
furanyl)ethyl]
decahydro-1-
naphthalenecar
boxylic acid

C20 H28 O4

14.87

332.1976

88.0

© Reported with Compound Discoverer 2.1

63

Compounds

19-Aug-2019 3:26

**Structure**

**Name**

**Formula**

**FISh
Coverage**

**RT [min]**

**Molecular Weight**

**mzCloud
Best Match**

**Group Areas**

**Area**

Neochlorogenic
acid

C16 H18 O9

4.62

354.0951

87.9

© Reported with Compound Discoverer 2.1

64

Compounds

19-Aug-2019 3:26

**Structure**

**Name**

**Formula**

**FISh
Coverage**

**RT [min]**

**Molecular Weight**

**mzCloud
Best Match**

**Group Areas**

**Area**

(3aR,4aS,5R,
7aS,8S,9aR)-5-
Hydroxy-4a,8-
dimethyl-3-
methyleneoctah
ydroazuleno[6,
5-b]furan-2,
6(3H,4H)-dione

C15 H20 O4

10.90

264.1354

87.7

© Reported with Compound Discoverer 2.1

65

Compounds

19-Aug-2019 3:26

**Structure**

**Name**

**Formula**

**FISh
Coverage**

**RT [min]**

**Molecular Weight**

**mzCloud
Best Match**

**Group Areas**

**Area**

1,4a-Dimethyl-
6-methylene-5-
[2-(2-oxo-2,5-
dihydro-3-
furanyl)ethyl]
decahydro-1-
naphthalenecar
boxylic acid

C20 H28 O4

11.19

332.1976

87.7

© Reported with Compound Discoverer 2.1

66

Compounds

19-Aug-2019 3:26

**Structure**

**Name**

**Formula**

**FISh
Coverage**

**RT [min]**

**Molecular Weight**

**mzCloud
Best Match**

**Group Areas**

**Area**

(+/-)12(13)-
DiHOME

C18 H34 O4

12.84

314.2459

87.6

© Reported with Compound Discoverer 2.1

67

Compounds

19-Aug-2019 3:26

**Structure**

**Name**

**Formula**

**FISh
Coverage**

**RT [min]**

**Molecular Weight**

**mzCloud
Best Match**

**Group Areas**

**Area**

3,5a,9-
Trimethyl-
2,3,3a,4,5,5a,8,
9b-
octahydronapht
ho[1,2-b]furan-
2,8-dione

C15 H18 O3

10.13

246.1250

87.5

© Reported with Compound Discoverer 2.1

68

Compounds

19-Aug-2019 3:26

**Structure**

**Name**

**Formula**

**FISh
Coverage**

**RT [min]**

**Molecular Weight**

**mzCloud
Best Match**

**Group Areas**

**Area**

Cafestol

C20 H28 O3

10.24

316.2026

87.5

© Reported with Compound Discoverer 2.1

69

Compounds

19-Aug-2019 3:26

**Structure**

**Name**

**Formula**

**FISh
Coverage**

**RT [min]**

**Molecular Weight**

**mzCloud
Best Match**

**Group Areas**

**Area**

(3aR,8R,8aR,
9aR)-8-
Hydroxy-8a-
methyl-3,5-
bis(methylene)
decahydronapht
ho[2,3-b]furan-
2(3H)-one

C15 H20 O3

7.96

248.1405

87.3

© Reported with Compound Discoverer 2.1

70

Compounds

19-Aug-2019 3:26

**Structure**

**Name**

**Formula**

**FISh
Coverage**

**RT [min]**

**Molecular Weight**

**mzCloud
Best Match**

**Group Areas**

**Area**

Medroxyprogest
erone

C22 H32 O3

13.88

344.2342

87.3

© Reported with Compound Discoverer 2.1

71

Compounds

19-Aug-2019 3:26

**Structure**

**Name**

**Formula**

**FISh
Coverage**

**RT [min]**

**Molecular Weight**

**mzCloud
Best Match**

**Group Areas**

**Area**

Chlorogenic
acid

C16 H18 O9

5.59

354.0951

87.3

© Reported with Compound Discoverer 2.1

72

Compounds

19-Aug-2019 3:26

**Structure**

**Name**

**Formula**

**FISh
Coverage**

**RT [min]**

**Molecular Weight**

**mzCloud
Best Match**

**Group Areas**

**Area**

Kahweol

C20 H26 O3

9.10

314.1871

87.2

© Reported with Compound Discoverer 2.1

73

Compounds

19-Aug-2019 3:26

**Structure**

**Name**

**Formula**

**FISh
Coverage**

**RT [min]**

**Molecular Weight**

**mzCloud
Best Match**

**Group Areas**

**Area**

11-
Ketotestosteron
e

C19 H26 O3

10.97

302.1875

87.1

© Reported with Compound Discoverer 2.1

74

Compounds

19-Aug-2019 3:26

**Structure**

**Name**

**Formula**

**FISh
Coverage**

**RT [min]**

**Molecular Weight**

**mzCloud
Best Match**

**Group Areas**

**Area**

Lariciresinol 4-
O-glucoside

C26 H34 O11

7.29

522.2103

87.1

© Reported with Compound Discoverer 2.1

75

Compounds

19-Aug-2019 3:26

**Structure**

**Name**

**Formula**

**FISh
Coverage**

**RT [min]**

**Molecular Weight**

**mzCloud
Best Match**

**Group Areas**

**Area**

1-[3-Hydroxy-2-
(2-hydroxy-2-
propanyl)-2,3-
dihydro-1-
benzofuran-5-
yl]ethanone

C13 H16 O4

8.37

236.1044

86.9

© Reported with Compound Discoverer 2.1

76

Compounds

19-Aug-2019 3:26

**Structure**

**Name**

**Formula**

**FISh
Coverage**

**RT [min]**

**Molecular Weight**

**mzCloud
Best Match**

**Group Areas**

**Area**

(3aS,10aR,
10bR)-6,10a-
Dimethyl-3-
methylene-
3,3a,4,5,7,8,
10a,10b-
octahydrofuro
[3',2':6,
7]cyclohepta[1,
2-b]pyran-2,9-
dione

C15 H18 O4

10.46

262.1197

86.7

© Reported with Compound Discoverer 2.1

77

Compounds

19-Aug-2019 3:26

**Structure**

**Name**

**Formula**

**FISh
Coverage**

**RT [min]**

**Molecular Weight**

**mzCloud
Best Match**

**Group Areas**

**Area**

© Reported with Compound Discoverer 2.1

78

Compounds

19-Aug-2019 3:26

**Structure**

**Name**

**Formula**

**FISh
Coverage**

**RT [min]**

**Molecular Weight**

**mzCloud
Best Match**

**Group Areas**

**Area**

D-(+)-Maltose

C12 H22 O11

1.09

364.0979

86.4

© Reported with Compound Discoverer 2.1

79

Compounds

19-Aug-2019 3:26

**Structure**

**Name**

**Formula**

**FISh
Coverage**

**RT [min]**

**Molecular Weight**

**mzCloud
Best Match**

**Group Areas**

**Area**

Linoleic acid

C18 H32 O2

17.56

280.2402

86.4

© Reported with Compound Discoverer 2.1

80

Compounds

19-Aug-2019 3:26

**Structure**

**Name**

**Formula**

**FISh
Coverage**

**RT [min]**

**Molecular Weight**

**mzCloud
Best Match**

**Group Areas**

**Area**

(15Z)-9,12,13-
Trihydroxy-15-
octadecenoic
acid

C18 H34 O5

10.51

330.2408

86.3

© Reported with Compound Discoverer 2.1

81

Compounds

19-Aug-2019 3:26

**Structure**

**Name**

**Formula**

**FISh
Coverage**

**RT [min]**

**Molecular Weight**

**mzCloud
Best Match**

**Group Areas**

**Area**

L-Phenylalanine

C9 H11 N O2

2.74

165.0790

86.2

© Reported with Compound Discoverer 2.1

82

Compounds

19-Aug-2019 3:26

**Structure**

**Name**

**Formula**

**FISh
Coverage**

**RT [min]**

**Molecular Weight**

**mzCloud
Best Match**

**Group Areas**

**Area**

Arachidonic acid

C20 H32 O2

13.38

286.2293

86.2

© Reported with Compound Discoverer 2.1

83

Compounds

19-Aug-2019 3:26

**Structure**

**Name**

**Formula**

**FISh
Coverage**

**RT [min]**

**Molecular Weight**

**mzCloud
Best Match**

**Group Areas**

**Area**

Testosterone

C19 H28 O2

11.95

288.2081

86.2

© Reported with Compound Discoverer 2.1

84

Compounds

19-Aug-2019 3:26

**Structure**

**Name**

**Formula**

**FISh
Coverage**

**RT [min]**

**Molecular Weight**

**mzCloud
Best Match**

**Group Areas**

**Area**

3,4-
Dimethoxycinna
mic acid

C11 H12 O4

8.77

208.0731

86.2

© Reported with Compound Discoverer 2.1

85

Compounds

19-Aug-2019 3:26

**Structure**

**Name**

**Formula**

**FISh
Coverage**

**RT [min]**

**Molecular Weight**

**mzCloud
Best Match**

**Group Areas**

**Area**

(-)-
Caryophyllene
oxide

C15 H24 O

15.39

220.1821

86.0

© Reported with Compound Discoverer 2.1

86

Compounds

19-Aug-2019 3:26

**Structure**

**Name**

**Formula**

**FISh
Coverage**

**RT [min]**

**Molecular Weight**

**mzCloud
Best Match**

**Group Areas**

**Area**

10-HDA

C10 H18 O3

11.49

168.1145

85.9

© Reported with Compound Discoverer 2.1

87

Compounds

19-Aug-2019 3:26

**Structure**

**Name**

**Formula**

**FISh
Coverage**

**RT [min]**

**Molecular Weight**

**mzCloud
Best Match**

**Group Areas**

**Area**

4-
Indolecarbaldeh
yde

C9 H7 N O

7.84

145.0528

85.9

© Reported with Compound Discoverer 2.1

88

Compounds

19-Aug-2019 3:26

**Structure**

**Name**

**Formula**

**FISh
Coverage**

**RT [min]**

**Molecular Weight**

**mzCloud
Best Match**

**Group Areas**

**Area**

Oleoyl
ethanolamide

C20 H39 N O2

17.96

307.2870

85.8

© Reported with Compound Discoverer 2.1

89

Compounds

19-Aug-2019 3:26

**Structure**

**Name**

**Formula**

**FISh
Coverage**

**RT [min]**

**Molecular Weight**

**mzCloud
Best Match**

**Group Areas**

**Area**

Phloroglucinol

C6 H6 O3

2.26

126.0317

85.8

© Reported with Compound Discoverer 2.1

90

Compounds

19-Aug-2019 3:26

**Structure**

**Name**

**Formula**

**FISh
Coverage**

**RT [min]**

**Molecular Weight**

**mzCloud
Best Match**

**Group Areas**

**Area**

Corchorifatty
acid F

C18 H32 O5

10.17

328.2253

85.6

© Reported with Compound Discoverer 2.1

91

Compounds

19-Aug-2019 3:26

**Structure**

**Name**

**Formula**

**FISh
Coverage**

**RT [min]**

**Molecular Weight**

**mzCloud
Best Match**

**Group Areas**

**Area**

Methyldienolone

C19 H26 O2

13.11

286.1924

85.5

© Reported with Compound Discoverer 2.1

92

Compounds

19-Aug-2019 3:26

**Structure**

**Name**

**Formula**

**FISh
Coverage**

**RT [min]**

**Molecular Weight**

**mzCloud
Best Match**

**Group Areas**

**Area**

6-
Hydroxynicotinic
acid

C6 H5 N O3

1.31

139.0266

85.3

© Reported with Compound Discoverer 2.1

93

Compounds

19-Aug-2019 3:26

**Structure**

**Name**

**Formula**

**FISh
Coverage**

**RT [min]**

**Molecular Weight**

**mzCloud
Best Match**

**Group Areas**

**Area**

Methyl
cinnamate

C10 H10 O2

14.64

162.0678

85.2

© Reported with Compound Discoverer 2.1

94

Compounds

19-Aug-2019 3:26

**Structure**

**Name**

**Formula**

**FISh
Coverage**

**RT [min]**

**Molecular Weight**

**mzCloud
Best Match**

**Group Areas**

**Area**

Androsterone

C19 H30 O2

10.70

272.2135

85.2

© Reported with Compound Discoverer 2.1

95

Compounds

19-Aug-2019 3:26

**Structure**

**Name**

**Formula**

**FISh
Coverage**

**RT [min]**

**Molecular Weight**

**mzCloud
Best Match**

**Group Areas**

**Area**

Myristyl sulfate

C14 H30 O4 S

27.06

294.1867

85.2

© Reported with Compound Discoverer 2.1

96

Compounds

19-Aug-2019 3:26

**Structure**

**Name**

**Formula**

**FISh
Coverage**

**RT [min]**

**Molecular Weight**

**mzCloud
Best Match**

**Group Areas**

**Area**

10-HDA

C10 H18 O3

7.09

186.1256

85.0

© Reported with Compound Discoverer 2.1

97

Compounds

19-Aug-2019 3:26

**Structure**

**Name**

**Formula**

**FISh
Coverage**

**RT [min]**

**Molecular Weight**

**mzCloud
Best Match**

**Group Areas**

**Area**

Arachidonic acid

C20 H32 O2

17.38

304.2392

85.0

© Reported with Compound Discoverer 2.1

98

Compounds

19-Aug-2019 3:26

**Structure**

**Name**

**Formula**

**FISh
Coverage**

**RT [min]**

**Molecular Weight**

**mzCloud
Best Match**

**Group Areas**

**Area**

4-Phenylbutyric
acid

C10 H12 O2

7.64

164.0836

84.8

© Reported with Compound Discoverer 2.1

99

Compounds

19-Aug-2019 3:26

**Structure**

**Name**

**Formula**

**FISh
Coverage**

**RT [min]**

**Molecular Weight**

**mzCloud
Best Match**

**Group Areas**

**Area**

Azelaic acid

C9 H16 O4

8.24

188.1041

84.8

© Reported with Compound Discoverer 2.1

100

Compounds

19-Aug-2019 3:26

**Structure**

**Name**

**Formula**

**FISh
Coverage**

**RT [min]**

**Molecular Weight**

**mzCloud
Best Match**

**Group Areas**

**Area**

(-)-
Caryophyllene
oxide

C15 H24 O

10.85

220.1821

84.6

© Reported with Compound Discoverer 2.1

101

Compounds

19-Aug-2019 3:26

**Structure**

**Name**

**Formula**

**FISh
Coverage**

**RT [min]**

**Molecular Weight**

**mzCloud
Best Match**

**Group Areas**

**Area**

Paracetamol

C8 H9 N O2

4.22

151.0632

84.3

© Reported with Compound Discoverer 2.1

102

Compounds

19-Aug-2019 3:26

**Structure**

**Name**

**Formula**

**FISh
Coverage**

**RT [min]**

**Molecular Weight**

**mzCloud
Best Match**

**Group Areas**

**Area**

1,4a-Dimethyl-
6-methylene-5-
[2-(2-oxo-2,5-
dihydro-3-
furanyl)ethyl]
decahydro-1-
naphthalenecar
boxylic acid

C20 H28 O4

9.08

332.1976

84.2

© Reported with Compound Discoverer 2.1

103

Compounds

19-Aug-2019 3:26

**Structure**

**Name**

**Formula**

**FISh
Coverage**

**RT [min]**

**Molecular Weight**

**mzCloud
Best Match**

**Group Areas**

**Area**

Prostaglandin
F2α 1-11-
lactone

C20 H32 O4

14.71

318.2193

84.2

© Reported with Compound Discoverer 2.1

104

Compounds

19-Aug-2019 3:26

**Structure**

**Name**

**Formula**

**FISh
Coverage**

**RT [min]**

**Molecular Weight**

**mzCloud
Best Match**

**Group Areas**

**Area**

7,8-
Bis
(hydroxymethyl)
-1,4a-dimethyl-
3,4,4a,5,6,7-
hexahydro-2H-
benzo[7]
annulen-2-one

C15 H22 O3

11.11

250.1561

84.1

© Reported with Compound Discoverer 2.1

105

Compounds

19-Aug-2019 3:26

**Structure**

**Name**

**Formula**

**FISh
Coverage**

**RT [min]**

**Molecular Weight**

**mzCloud
Best Match**

**Group Areas**

**Area**

6:2 Fluorinated
telomer
sulfonate

C8 H5 F13 O3 S

13.79

427.9751

84.0

© Reported with Compound Discoverer 2.1

106

Compounds

19-Aug-2019 3:26

**Structure**

**Name**

**Formula**

**FISh
Coverage**

**RT [min]**

**Molecular Weight**

**mzCloud
Best Match**

**Group Areas**

**Area**

Dehydroepiandr
osterone
(DHEA)

C19 H28 O2

13.66

270.1977

84.0

© Reported with Compound Discoverer 2.1

107

Compounds

19-Aug-2019 3:26

**Structure**

**Name**

**Formula**

**FISh
Coverage**

**RT [min]**

**Molecular Weight**

**mzCloud
Best Match**

**Group Areas**

**Area**

DL-Stachydrine

C7 H13 N O2

1.17

143.0946

84.0

© Reported with Compound Discoverer 2.1

108

Compounds

19-Aug-2019 3:26

**Structure**

**Name**

**Formula**

**FISh
Coverage**

**RT [min]**

**Molecular Weight**

**mzCloud
Best Match**

**Group Areas**

**Area**

5,7-Dihydroxy-
4-
methylcoumarin

C10 H8 O4

6.18

192.0421

84.0

© Reported with Compound Discoverer 2.1

109

Compounds

19-Aug-2019 3:26

**Structure**

**Name**

**Formula**

**FISh
Coverage**

**RT [min]**

**Molecular Weight**

**mzCloud
Best Match**

**Group Areas**

**Area**

Dehydroepiandr
osterone
(DHEA)

C19 H28 O2

14.12

270.1977

83.9

© Reported with Compound Discoverer 2.1

110

Compounds

19-Aug-2019 3:26

**Structure**

**Name**

**Formula**

**FISh
Coverage**

**RT [min]**

**Molecular Weight**

**mzCloud
Best Match**

**Group Areas**

**Area**

Verrucarol

C15 H22 O4

7.96

266.1513

83.9

© Reported with Compound Discoverer 2.1

111

Compounds

19-Aug-2019 3:26

**Structure**

**Name**

**Formula**

**FISh
Coverage**

**RT [min]**

**Molecular Weight**

**mzCloud
Best Match**

**Group Areas**

**Area**

Cyclo
(leucylprolyl)

C11 H18 N2 O2

6.86

210.1366

83.7

© Reported with Compound Discoverer 2.1

112

Compounds

19-Aug-2019 3:26

**Structure**

**Name**

**Formula**

**FISh
Coverage**

**RT [min]**

**Molecular Weight**

**mzCloud
Best Match**

**Group Areas**

**Area**

Prostaglandin
F2α 1-11-
lactone

C20 H32 O4

13.65

318.2194

83.7

© Reported with Compound Discoverer 2.1

113

Compounds

19-Aug-2019 3:26

**Structure**

**Name**

**Formula**

**FISh
Coverage**

**RT [min]**

**Molecular Weight**

**mzCloud
Best Match**

**Group Areas**

**Area**

Etiocholanolone

C19 H30 O2

12.37

272.2135

83.7

© Reported with Compound Discoverer 2.1

114

Compounds

19-Aug-2019 3:26

**Structure**

**Name**

**Formula**

**FISh
Coverage**

**RT [min]**

**Molecular Weight**

**mzCloud
Best Match**

**Group Areas**

**Area**

3-(4-
Methylbenzoyl)
acrylic acid

C11 H10 O3

10.22

190.0624

83.7

© Reported with Compound Discoverer 2.1

115

Compounds

19-Aug-2019 3:26

**Structure**

**Name**

**Formula**

**FISh
Coverage**

**RT [min]**

**Molecular Weight**

**mzCloud
Best Match**

**Group Areas**

**Area**

5,2'-Dihydroxy-
6,7,8,6'-
tetramethoxyflav
one

C19 H18 O8

11.57

374.0991

83.6

© Reported with Compound Discoverer 2.1

116

Compounds

19-Aug-2019 3:26

**Structure**

**Name**

**Formula**

**FISh
Coverage**

**RT [min]**

**Molecular Weight**

**mzCloud
Best Match**

**Group Areas**

**Area**

Vanillin

C8 H8 O3

6.66

152.0474

83.5

© Reported with Compound Discoverer 2.1

117

Compounds

19-Aug-2019 3:26

**Structure**

**Name**

**Formula**

**FISh
Coverage**

**RT [min]**

**Molecular Weight**

**mzCloud
Best Match**

**Group Areas**

**Area**

1-
(Carboxymethyl)
cyclohexanecar
boxylic acid

C9 H14 O4

6.51

186.0885

83.5

© Reported with Compound Discoverer 2.1

118

Compounds

19-Aug-2019 3:26

**Structure**

**Name**

**Formula**

**FISh
Coverage**

**RT [min]**

**Molecular Weight**

**mzCloud
Best Match**

**Group Areas**

**Area**

(15Z)-9,12,13-
Trihydroxy-15-
octadecenoic
acid

C18 H34 O5

11.30

330.2408

83.4

© Reported with Compound Discoverer 2.1

119

Compounds

19-Aug-2019 3:26

**Structure**

**Name**

**Formula**

**FISh
Coverage**

**RT [min]**

**Molecular Weight**

**mzCloud
Best Match**

**Group Areas**

**Area**

(1R,4aS)-7-(2-
Hydroxypropan-
2-yl)-1,4a-
dimethyl-9-oxo-
3,4,10,10a-
tetrahydro-2H-
phenanthrene-
1-carboxylic
acid

C20 H26 O4

12.70

330.1820

83.4

© Reported with Compound Discoverer 2.1

120

Compounds

19-Aug-2019 3:26

**Structure**

**Name**

**Formula**

**FISh
Coverage**

**RT [min]**

**Molecular Weight**

**mzCloud
Best Match**

**Group Areas**

**Area**

Prostaglandin
F2α 1-11-
lactone

C20 H32 O4

13.41

318.2194

83.4

© Reported with Compound Discoverer 2.1

121

Compounds

19-Aug-2019 3:26

**Structure**

**Name**

**Formula**

**FISh
Coverage**

**RT [min]**

**Molecular Weight**

**mzCloud
Best Match**

**Group Areas**

**Area**

Cyclo
(leucylprolyl)

C11 H18 N2 O2

6.59

210.1366

83.4

© Reported with Compound Discoverer 2.1

122

Compounds

19-Aug-2019 3:26

**Structure**

**Name**

**Formula**

**FISh
Coverage**

**RT [min]**

**Molecular Weight**

**mzCloud
Best Match**

**Group Areas**

**Area**

Glycerophospho
-N-palmitoyl
ethanolamine

C21 H44 N O7 P

27.19

453.2860

83.3

© Reported with Compound Discoverer 2.1

123

Compounds

19-Aug-2019 3:26

**Structure**

**Name**

**Formula**

**FISh
Coverage**

**RT [min]**

**Molecular Weight**

**mzCloud
Best Match**

**Group Areas**

**Area**

L-Norleucine

C6 H13 N O2

1.58

131.0947

83.3

© Reported with Compound Discoverer 2.1

124

Compounds

19-Aug-2019 3:26

**Structure**

**Name**

**Formula**

**FISh
Coverage**

**RT [min]**

**Molecular Weight**

**mzCloud
Best Match**

**Group Areas**

**Area**

Gluconic acid

C6 H12 O7

1.55

196.0576

83.2

© Reported with Compound Discoverer 2.1

125

Compounds

19-Aug-2019 3:26

**Structure**

**Name**

**Formula**

**FISh
Coverage**

**RT [min]**

**Molecular Weight**

**mzCloud
Best Match**

**Group Areas**

**Area**

Glycerophospho
-N-palmitoyl
ethanolamine

C21 H44 N O7 P

27.44

453.2856

83.1

© Reported with Compound Discoverer 2.1

126

Compounds

19-Aug-2019 3:26

**Structure**

**Name**

**Formula**

**FISh
Coverage**

**RT [min]**

**Molecular Weight**

**mzCloud
Best Match**

**Group Areas**

**Area**

N-Acetyl-D-
alloisoleucine

C8 H15 N O3

6.35

173.1044

83.0

© Reported with Compound Discoverer 2.1

127

Compounds

19-Aug-2019 3:26

**Structure**

**Name**

**Formula**

**FISh
Coverage**

**RT [min]**

**Molecular Weight**

**mzCloud
Best Match**

**Group Areas**

**Area**

Prostaglandin
F2α 1-11-
lactone

C20 H32 O4

12.62

318.2194

83.0

© Reported with Compound Discoverer 2.1

128

Compounds

19-Aug-2019 3:26

**Structure**

**Name**

**Formula**

**FISh
Coverage**

**RT [min]**

**Molecular Weight**

**mzCloud
Best Match**

**Group Areas**

**Area**

Avocadyne 1-
acetate

C19 H34 O4

17.17

308.2342

83.0

© Reported with Compound Discoverer 2.1

129

Compounds

19-Aug-2019 3:26

**Structure**

**Name**

**Formula**

**FISh
Coverage**

**RT [min]**

**Molecular Weight**

**mzCloud
Best Match**

**Group Areas**

**Area**

(15Z)-9,12,13-
Trihydroxy-15-
octadecenoic
acid

C18 H34 O5

11.03

330.2408

82.8

© Reported with Compound Discoverer 2.1

130

Compounds

19-Aug-2019 3:26

**Structure**

**Name**

**Formula**

**FISh
Coverage**

**RT [min]**

**Molecular Weight**

**mzCloud
Best Match**

**Group Areas**

**Area**

Ambrosic acid

C15 H20 O4

10.90

281.1621

82.8

© Reported with Compound Discoverer 2.1

131

Compounds

19-Aug-2019 3:26

**Structure**

**Name**

**Formula**

**FISh
Coverage**

**RT [min]**

**Molecular Weight**

**mzCloud
Best Match**

**Group Areas**

**Area**

Verrucarol

C15 H22 O4

8.24

266.1513

82.8

© Reported with Compound Discoverer 2.1

132

Compounds

19-Aug-2019 3:26

**Structure**

**Name**

**Formula**

**FISh
Coverage**

**RT [min]**

**Molecular Weight**

**mzCloud
Best Match**

**Group Areas**

**Area**

Dibutyl
phthalate

C16 H22 O4

13.99

278.1519

82.8

© Reported with Compound Discoverer 2.1

133

Compounds

19-Aug-2019 3:26

**Structure**

**Name**

**Formula**

**FISh
Coverage**

**RT [min]**

**Molecular Weight**

**mzCloud
Best Match**

**Group Areas**

**Area**

Nootkatone

C15 H22 O

9.98

218.1665

82.5

© Reported with Compound Discoverer 2.1

134

Compounds

19-Aug-2019 3:26

**Structure**

**Name**

**Formula**

**FISh
Coverage**

**RT [min]**

**Molecular Weight**

**mzCloud
Best Match**

**Group Areas**

**Area**

Leucine

C6 H13 N O2

1.09

131.0947

82.5

© Reported with Compound Discoverer 2.1

135

Compounds

19-Aug-2019 3:26

**Structure**

**Name**

**Formula**

**FISh
Coverage**

**RT [min]**

**Molecular Weight**

**mzCloud
Best Match**

**Group Areas**

**Area**

(+/-)12(13)-
DiHOME

C18 H34 O4

14.17

296.2352

82.4

© Reported with Compound Discoverer 2.1

136

Compounds

19-Aug-2019 3:26

**Structure**

**Name**

**Formula**

**FISh
Coverage**

**RT [min]**

**Molecular Weight**

**mzCloud
Best Match**

**Group Areas**

**Area**

Suberic acid

C8 H14 O4

7.13

174.0884

82.4

© Reported with Compound Discoverer 2.1

137

Compounds

19-Aug-2019 3:26

**Structure**

**Name**

**Formula**

**FISh
Coverage**

**RT [min]**

**Molecular Weight**

**mzCloud
Best Match**

**Group Areas**

**Area**

10-HDA

C10 H18 O3

7.66

168.1145

82.4

© Reported with Compound Discoverer 2.1

138

Compounds

19-Aug-2019 3:26

**Structure**

**Name**

**Formula**

**FISh
Coverage**

**RT [min]**

**Molecular Weight**

**mzCloud
Best Match**

**Group Areas**

**Area**

Norharman

C11 H8 N2

6.00

168.0685

82.3

© Reported with Compound Discoverer 2.1

139

Compounds

19-Aug-2019 3:26

**Structure**

**Name**

**Formula**

**FISh
Coverage**

**RT [min]**

**Molecular Weight**

**mzCloud
Best Match**

**Group Areas**

**Area**

3-Hydroxy-2-
methylpyridine

C6 H7 N O

6.50

109.0531

82.3

© Reported with Compound Discoverer 2.1

140

Compounds

19-Aug-2019 3:26

**Structure**

**Name**

**Formula**

**FISh
Coverage**

**RT [min]**

**Molecular Weight**

**mzCloud
Best Match**

**Group Areas**

**Area**

3-(1-
hydroxyethyl)-
2,3,6,7,8,8a-
hexahydropyrrol
o[1,2-
a]pyrazine-1,4-
dione

C9 H14 N2 O3

2.52

198.1003

82.1

© Reported with Compound Discoverer 2.1

141

Compounds

19-Aug-2019 3:26

**Structure**

**Name**

**Formula**

**FISh
Coverage**

**RT [min]**

**Molecular Weight**

**mzCloud
Best Match**

**Group Areas**

**Area**

Citrinin

C13 H14 O5

12.46

232.0729

82.0

© Reported with Compound Discoverer 2.1

142

Compounds

19-Aug-2019 3:26

**Structure**

**Name**

**Formula**

**FISh
Coverage**

**RT [min]**

**Molecular Weight**

**mzCloud
Best Match**

**Group Areas**

**Area**

Eucalyptol

C10 H18 O

8.28

136.1250

81.9

© Reported with Compound Discoverer 2.1

143

Compounds

19-Aug-2019 3:26

**Structure**

**Name**

**Formula**

**FISh
Coverage**

**RT [min]**

**Molecular Weight**

**mzCloud
Best Match**

**Group Areas**

**Area**

Isotretinoin

C20 H28 O2

12.43

300.2080

81.9

© Reported with Compound Discoverer 2.1

144

Compounds

19-Aug-2019 3:26

**Structure**

**Name**

**Formula**

**FISh
Coverage**

**RT [min]**

**Molecular Weight**

**mzCloud
Best Match**

**Group Areas**

**Area**

Isotretinoin

C20 H28 O2

16.49

300.2080

81.9

© Reported with Compound Discoverer 2.1

145

Compounds

19-Aug-2019 3:26

**Structure**

**Name**

**Formula**

**FISh
Coverage**

**RT [min]**

**Molecular Weight**

**mzCloud
Best Match**

**Group Areas**

**Area**

15-Deoxy-
δ12,14 -
Prostaglandin
J2

C20 H28 O3

14.01

316.2038

81.8

© Reported with Compound Discoverer 2.1

146

Compounds

19-Aug-2019 3:26

**Structure**

**Name**

**Formula**

**FISh
Coverage**

**RT [min]**

**Molecular Weight**

**mzCloud
Best Match**

**Group Areas**

**Area**

DL-Stachydrine

C7 H13 N O2

1.43

143.0946

81.8

© Reported with Compound Discoverer 2.1

147

Compounds

19-Aug-2019 3:26

**Structure**

**Name**

**Formula**

**FISh
Coverage**

**RT [min]**

**Molecular Weight**

**mzCloud
Best Match**

**Group Areas**

**Area**

Isotretinoin

C20 H28 O2

15.93

300.2080

81.7

© Reported with Compound Discoverer 2.1

148

Compounds

19-Aug-2019 3:26

**Structure**

**Name**

**Formula**

**FISh
Coverage**

**RT [min]**

**Molecular Weight**

**mzCloud
Best Match**

**Group Areas**

**Area**

Prostaglandin
F2α 1-11-
lactone

C20 H32 O4

12.97

318.2190

81.7

© Reported with Compound Discoverer 2.1

149

Compounds

19-Aug-2019 3:26

**Structure**

**Name**

**Formula**

**FISh
Coverage**

**RT [min]**

**Molecular Weight**

**mzCloud
Best Match**

**Group Areas**

**Area**

© Reported with Compound Discoverer 2.1

150
